# Structural insight into the mechanism of stabilization of the 7SK small nuclear RNA by LARP7

**DOI:** 10.1093/nar/gkv173

**Published:** 2015-03-09

**Authors:** Emiko Uchikawa, Kundhavai S. Natchiar, Xiao Han, Florence Proux, Pierre Roblin, Elodie Zhang, Alexandre Durand, Bruno P. Klaholz, Anne-Catherine Dock-Bregeon

**Affiliations:** 1 Department of Integrated Structural Biology, Centre for Integrative Biology (CBI), IGBMC (Institute of Genetics and of Molecular and Cellular Biology, 67404 Illkirch, France; 2 Centre National de la Recherche Scientifique (CNRS) UMR 7104, Illkirch, France; 3 Institut National de la Santé et de la Recherche Médicale (INSERM) U964, Illkirch, France; 4 Université de Strasbourg, 67000 Strasbourg, France; 5 Department of functional genomics, Institut de Biologie de l'Ecole Normale Supérieure (IBENS), 75005 Paris, France; 6 CNRS UMR 8197, 75005 Paris, France; 7 INSERM U1024, 75005 Paris, France; 8 Key Laboratory of Brain Functional Genomics, East China Normal University (ECNU), 200241 Shanghai, PR China; 9 SOLEIL Synchrotron, 91192 Gif-sur-Yvette, France; 10 INRA-URBIA, 44316 Nantes, France

## Abstract

The non-coding RNA 7SK is the scaffold for a small nuclear ribonucleoprotein (7SKsnRNP) which regulates the function of the positive transcription elongation factor P-TEFb in the control of RNA polymerase II elongation in metazoans. The La-related protein LARP7 is a component of the 7SKsnRNP required for stability and function of the RNA. To address the function of LARP7 we determined the crystal structure of its La module, which binds a stretch of uridines at the 3′-end of 7SK. The structure shows that the penultimate uridine is tethered by the two domains, the La-motif and the RNA-recognition motif (RRM1), and reveals that the RRM1 is significantly smaller and more exposed than in the La protein. Sequence analysis suggests that this impacts interaction with 7SK. Binding assays, footprinting and small-angle scattering experiments show that a second RRM domain located at the C-terminus binds the apical loop of the 3′ hairpin of 7SK, while the N-terminal domains bind at its foot. Our results suggest that LARP7 uses both its N- and C-terminal domains to stabilize 7SK in a closed structure, which forms by joining conserved sequences at the 5′-end with the foot of the 3′ hairpin and has thus functional implications.

## INTRODUCTION

The La-related proteins (LARPs) are involved in various important functions in RNA metabolism and are found in nearly all eukaryotes (1). Besides the essential role of the paradigmatic La protein in tRNA processing, and its involvement in transcription termination by binding to nascent transcripts generated by polymerase III (2), members of the LARP family are involved in the regulation of translation or demonstrate chaperoning activities (3). In addition to the characteristic domain containing the La-motif (LAM), related to the winged-helix domain, they possess several RNA-binding domains akin to the RNA recognition motif (RRM) structural fold (4). LARPs share a conserved two-domain unit, called the La module, comprising the LaM and RRM1. High-resolution structures of the La module have been described in the case of HsLa, the human La protein (5,6), and very recently for human LARP6 (7). As such, LARPs are modular proteins, with intriguing possibilities for intricate RNA-binding combinations. LARP7 is the family member showing the highest sequence similarity to La, with the characteristic La module in the N-terminal third of the protein (3,8). However, while La binds all nascent transcripts synthesized by RNA polymerase III via their shared termination motif, UUU_OH_, LARP7 binds almost exclusively to the non-coding RNA 7SK (9–11). In Drosophila, LARP7 and the members of the 7SK snRNP have recently been identified (12,13). Other potential LARP7 homologs are found in ciliates, such as P65 in *Tetrahymena thermophila*, which has been found to assist in the correct folding of the telomerase RNA and hierarchical assembly of the RNP (14).

**Figure 1.**
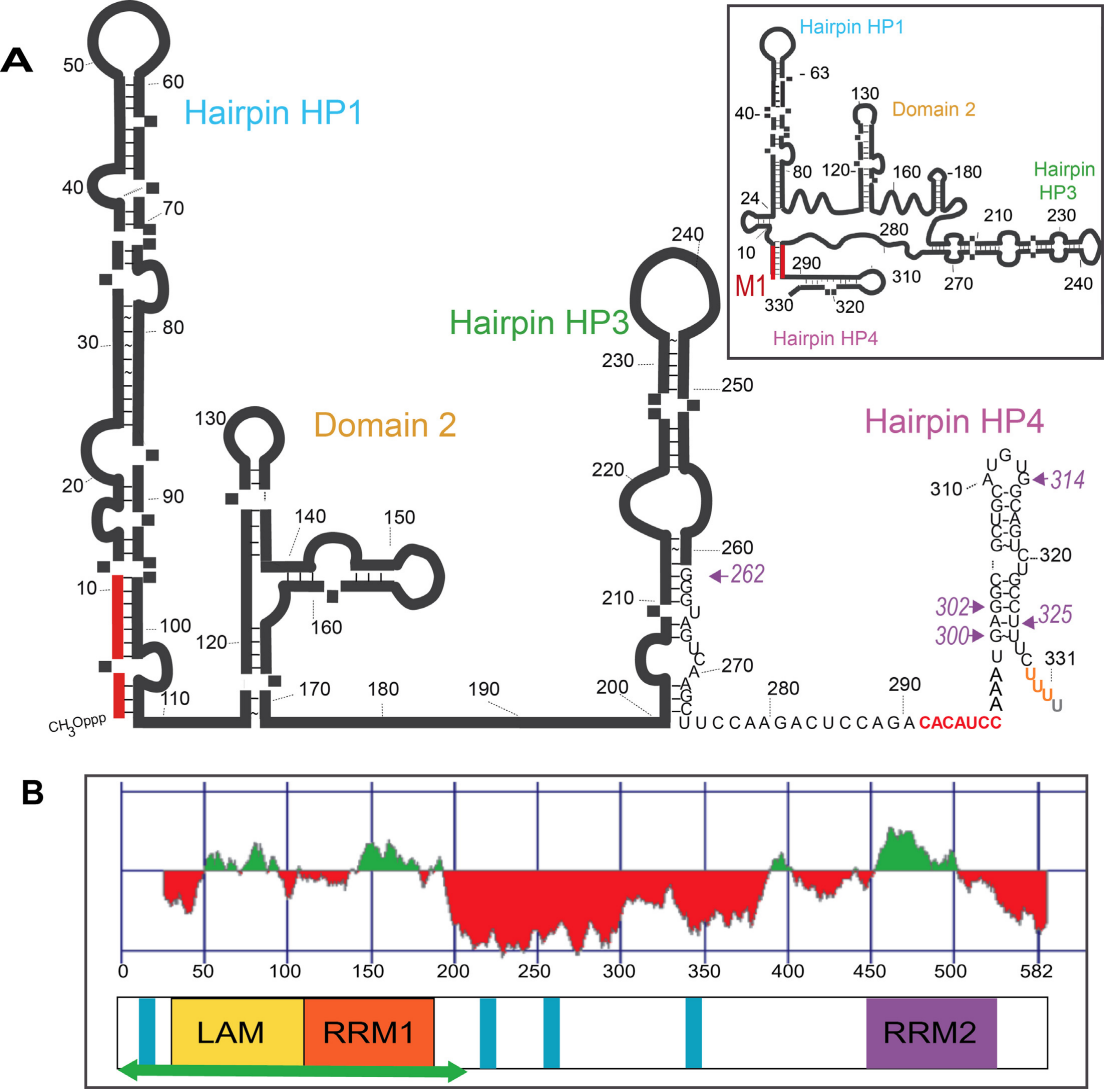
Domain organization of the molecules. (**A**) 2D model of 7SK with the 3′ U-triplet in orange and the additional residue 332 in gray. The two sequences that can form seven base-pairs are in red. The inset shows the closed 2D-structure thus formed. Arrows and numbers in purple indicate the 5′-boundary of the RNAs used in this work, for which the sequence is given. (**B**) Domain organization of LARP7 (582 amino acids in human) in linear representation with the color scheme adopted for the manuscript: LAM (28–111) yellow, RRM1 (120–188) orange and RRM2 (450–545) purple. The green horizontal arrow corresponds to the protein produced for the structural study. Blue bars represent stretches of basic residues. On top is the prediction of structure formation (red for unfolded, green for folded) as computed with Foldindex.

Although it was one of the first identified, 7SK still stands as an intriguing member among the fast-growing family of non-coding RNAs identified in humans (15,16). This abundant RNA found in the nucleus of higher eukaryotes functions as a regulator of P-TEFb, a transcription elongation factor required for the transition of promoter proximal paused polymerases into productive elongation (17–19), which is instrumental in regulating transcription in an appropriate temporal and spacial manner (20,21). 7SK sequesters and inactivates P-TEFb through the function of HEXIM proteins. Binding to 7SK enables HEXIM to interact with P-TEFb and inhibit its kinase activity (22–27). 7SK is a 331 nucleotide RNA transcribed by RNA polymerase III (Figure 1A). It has the usual stretch of uridines at the 3′-end that are required for efficient termination by RNA polymerase III. The 7SK-specific 5′ cap is mono-methylated at the gamma phosphate of the 5′ triphosphate by another component of the 7SKsnRNP, MePCE (also called BCDIN3 in Drosophila) (10). Together, MePCE and LARP7 bind 7SK on both ends, thus forming a stable 7SKsnRNP core protecting the RNA from exonucleases (9,11,28–29). A model for the 7SK 2D structure (Figure 1A), based on experimental probing data, was proposed in the early 90's (30), but while RNA domains involved in HEXIM-binding or P-TEFb regulation could be delineated according to it (31–35), it provided only poor information about how 7SK coordinates P-TEFb inhibition. Alternative 2D models for 7SK can be drawn with equivalent stabilities, suggesting that 7SK is intrinsically able to switch conformation. In fact, the original structural data can best be explained by the existence of at least two different conformations in the population of 7SK snRNPs in cells (36). An interesting model by Marz *et al*. (37) proposed the formation of a closed form of 7SK, based on the evolutionary conservation of sequences that would allow pairing of the first seven nucleotides of 7SK with a region just upstream of the terminal stem-loop (Figure 1A). This results in a lariat, a closed form with a dangling 3′-hairpin. In addition, this analysis highlighted the co-evolution of 7SK and LARP7, thus suggesting that LARP7 may have a chaperoning function for 7SK.

Several investigations carried out on human diseases highlighted the important role of LARP7 on the stability of the 7SK RNA, and consequently its function. Several frameshift mutations in LARP7 have been associated with gastric cancer (38). Mutations in the LARP7 gene were found associated with recessive cognitive disorders (39) and in primordial dwarfism associated with intellectual disability (40). These mutations seem to induce the loss of LARP7 protein through nonsense-mediated decay. Importantly, the loss of 7SK RNA as a consequence of the mutation was demonstrated in lymphoblasts from patients (40). This is in-line with previous experiments showing that the knockdown of LARP7 leads to decrease the level of nuclear 7SK in HeLa (11) and HEK293 cells (9).

As a consequence of the sequence similarity with La proteins, it was soon proposed that LARP7 uses its La module to bind the poly-uridine sequence at the 3′-end of 7SK (9,11). Indeed, almost half of the La module, the LAM region may be replaced with the LAM region of the genuine La protein, without dramatically reducing the binding to 7SK (11). In contrast, the specific recognition of 7SK involves two RRMs, the one adjacent to the LAM region (RRM1) and a C-terminal RRM (RRM2). This is demonstrated by the loss of binding specificity when the RRM1 is swapped with the RRM1 of La, or a point mutation introduced at a signature residue of RRMs (11).

To address the function of LARP7, we investigated the origin of LARP7 specificity for 7SK. The crystal structure of the La-homology domain of human LARP7, comprising LAM and RRM1, highlights specific features of the LARP7 RRM1 domain which suggest why it cannot be swapped for La RRM. The C-terminal RRM2 was shown by a combination of methods including binding assays, RNA footprinting and small-angle X-ray scattering (SAXS) to bind the apical loop of the 3′-hairpin. Taken together, our data support a model where both structural domains of LARP7 are combined to bind 7SK. LARP7 wrapping around the 3′ region includes the sequences closing the lariat form of 7SK. This constitutes a first evidence for the closed conformation of 7SK predicted by computational and phylogenetic analyses (37). LARP7 function would be to stabilize this closed conformation, thus bringing together the functional subdomains of 7SK.

## MATERIALS AND METHODS

### Preparation of RNAs and proteins

Several RNAs and protein constructs were designed, as detailed in the Supplementary Material section. All RNAs (apart from the 8-mer oligonucleotide UUUCUUUU, synthetic, from Dharmacon) were obtained by *in vitro* transcription. LARP7 full-length and the truncated versions were expressed in *Escherichia coli*. Mutagenesis was performed by the Quikchange approach (Stratagene).

### Crystallization of complexes with RNA

Plate-shaped crystals were obtained with 30% PEG 3350 and 0.1-M succinic acid, pH 7.0 at 4°C with several RNAs (detailed in Supplementary material) but very few crystals diffracted well. All RNAs leading to crystal formation comprised the 7SK 3′-end oligonucleotide 325–332, but not systematically the HP4 hairpin. The structure was solved from a crystal obtained in a drop initially set up with RNA 300–332 comprising the HP4 hairpin but gel analysis of the drop showed that the RNA was degraded. After structural analysis, it appeared that the largest piece of RNA bound to the protein was a 5-mer corresponding to the 3′-end, which was probably protected from degradation by binding to the protein.

### Crystal structure

The diffraction data were collected on beamline PX II on a Pilatus detector at SLS. The diffraction images were indexed and integrated using MOSFLM (41). The unmerged reflections were merged using the program SCALA (42) as a part of CCP4 suite of programs (43). The crystals belong to the space group C2 with the cell parameter *a* = 163.452 Å, *b* = 33.50 Å, *c* = 119.08 Å, *α* = 90.0°, *β* = 128.99° and *γ* = 90.0°. The structure solution was obtained by molecular replacement, using Phaser (44,45) with La protein (PDB 2VOO) as a search model (6). Initial rigid body and positional restraint refinement were carried out using CCP4 suite of programs (43). In the subsequent cycles, positional and B-factor refinements were performed using BUSTER and the simulated annealing refinements were carried out using CNS (46,47). Model building was carried out using COOT (48). Finally, the structure converged with R-factor and free R, 22.1 and 27.4%, respectively, with reasonable geometric parameters and B-factor (see Table 1 for statistics).

**Table 1.** Data collection and refinement statistics

Resolution range (Å)	92.58–3.2 (3.37–3.2)
Space group	C2
*a* (Å)	163.45
*b* (Å)	33.50
*c* (Å)	119.08
*β* (º)	128.99
R-merge	0.163 (1.034)
*I*/*σ*	5.41 (2.0)
Total number of reflections	27542 (3954)
Number of unique reflections	8165 (1171)
Completeness (%)	95.2% (94.9%)
Multiplicity	3.4 (3.4)
Refinement statistics	
Resolution limits (Å)	59.06–3.2 (3.58–3.2)
No. of reflections	8155
Protein atoms	2522
Nucleic acid atoms	168
Water molecules	5
Average B-value for all atoms (Å^2^)	93.7
R-factor (%)	22.1 (25.9)
R-free (%)	27.4 (30.8)
RMS deviation from ideal values in	
Bond length	0.01
Bond angles	1.32
Torsion angles	24.14
Peptide omega torsion angles	2.73
Ramachandran statistics	
Favored (%)	80.7
Allowed (%)	18.3
Outliers (%)	1.0

Last resolution shell details are given in paratheses.

### Binding assessment with electrophoretic mobility shift assays

The ϒ-^32^P-5′-labeled RNA (50 nM in all assays) was incubated 20 min at 4°C with increasing concentrations of proteins in a buffer containing 250-mM NaCl. Native gel analysis was performed as detailed in the Supplementary Material section.

### Footprinting

The 5′-labeled 262-HP4 RNAs (50 nM) were mixed with protein in a similar buffer as in electrophoretic mobility shift assay (EMSA) and incubated at 4°C. The concentrations of proteins were chosen to ensure 100% binding (1 μM for full-length, 2 μM for N- or C-terminal domains). The RNases concentrations were chosen to produce significant cleavages in 5 min, as detailed in the Supplementary Material section.

### Sequence alignments and figures

Two multiple sequence alignments were obtained independently. The first, for LARP7, resulted from a BLAST search starting with the human sequences Q4G0J3. Sequences were examined in the C-terminal region to distinguish LARP7 from other LARP sequences. The process led to about 50 sequences, treated with ClustalOmega, before visualization with Pymol (Figure 3B). The second alignment, of LARP7 and La, results from alignment with Muscle (49) of 15 sequences each, from species chosen to match as much as possible those presented in previous publications (1,50). The extracts presented in Supplementary Figures S2 and S7 were drawn with ESPRIPT (51). The figures were drawn using Pymol (The PyMOL Molecular Graphics System, Version 1.5.0.4 Schrödinger, LLC).

**Figure 2.**
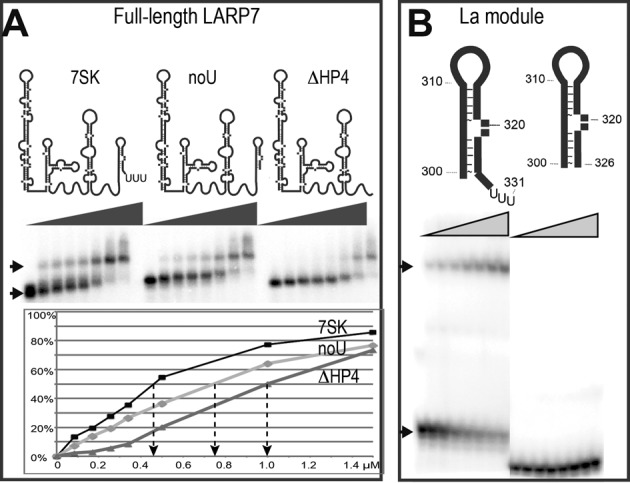
LARP7 N-terminal domain requires the 3′-end uridines for binding. (**A**) EMSA on a native agarose gel showing complex formation (arrow) after incubation of LARP7 full-length with 7SK: (7SK) wild-type (noU) RNA (1–328) deprived of the terminal U-triplet (ΔHP4) (1–295) deprived of the 3′-hairpin HP4. Complex quantification as a function of protein concentration is reported below. (**B**) Native gel after incubation with the La module (1–208) showing the free (lower arrow) and the complexed (top arow) RNAs, which are schematized on top: (HP4) hairpin 300–331, (HP4noU) hairpin 300–326 deprived of the terminal U-triplet.

**Figure 3.**
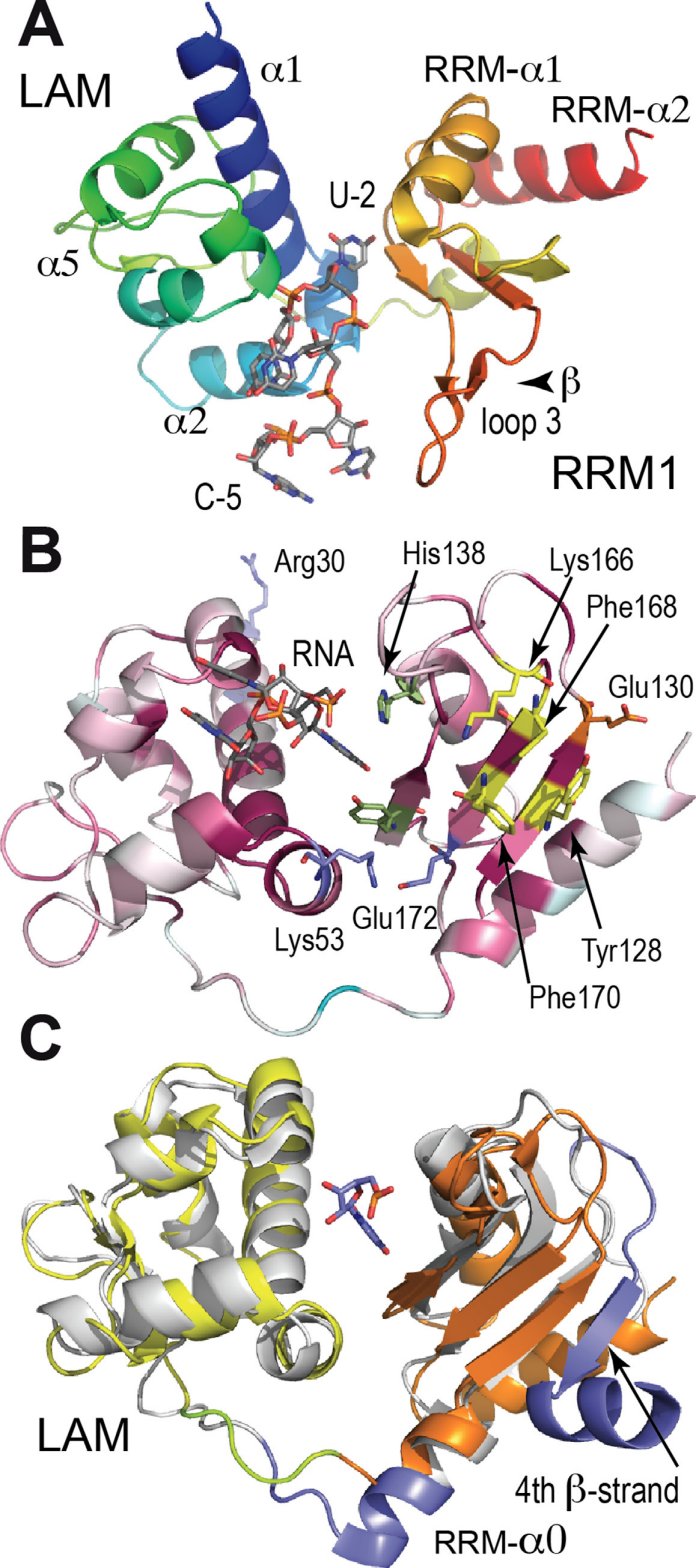
Global view of LARP7-Ndom and comparison with La. (**A**) The La module of LARP7 (LAM domain blue to green, RRM yellow to red) with the RNA in stick representation (gray). (**B**) Sequence conservation reported in the 3D structure, with ribbon colored from cyan (variable) to wine-red (conserved) and signature residues of RRMs in yellow. LARP7-invariants are highlighted in stick representation: Arg30, Lys32, Lys53, Glu172 (blue), Glu130 (orange), Tyr153, His138 in green. The uridine triplet is shown in gray. (**C**) Superposition of La (gray) and LARP7 (LAM, yellow and RRM1, orange) with the sequences absent in LARP7 highlighted in blue. U-2, here from LARP7 is at the same position in the two structures.

### Molecular modeling

The starting model of the extended M1-HP4 RNA bound to the C-terminal domain was obtained as follows. First, a model for the RNA M1-HP4 was created from a linear sequence comprising the 5′-nucleotides (residues 1–8 of 7SK) linked with sequence AGA to the sequence 289–328 (comprising HP4 without the terminal uridine triplet). This AGA, adjacent to G8, was expected to favor a GAGA tetraloop, thus folding the M1 extension into an independent hairpin of seven base-pairs. This linear sequence was submitted to MC-fold (52), which proposed several 3D models of the M1-HP4. Those were indeed composed of two hairpins (M1 and HP4) whose orientations varied mostly at the level of the linker sequence AAAU (296–299). A series of 3D models, representing the most divergent families, were finally obtained by replacing the coordinates of the HP4 hairpin by those extracted from the solution structure (53) and removal of the helical extension introduced by the authors. Models for the RNA M1-HP4 in complex with the C-terminal domain were then manually assembled with P65, without attempting to change the coordinates of the protein (PDB id. 4ERD), as this structure has been suggested to be similar to the the structure of LARP7 C-terminal domain (54).

### SAXS experiments

SAXS experiments were conducted on the SWING beamline at the SOLEIL synchrotron (*λ* = 1.033 Å). The Aviex charge-coupled device detector was positioned to collect data in the *Q*-range 0.008–0.33 4 Å−1 (*Q* = 4πsinθ λ−1, where 2θ is the scattering angle). All solutions were mixed in a fixed-temperature (15°C) quartz capillary with a diameter of 1.5 mm and a wall thickness of 10 μm, positioned within a vacuum chamber. Fifty microliter of a monodisperse sample of RNA–protein complex (70–130 μM) was injected onto a size-exclusion column (SEC-3 300, 150-Å Agilent), using an Agilent HPLC system, and eluted directly into the SAXS flow-through capillary cell at a flow rate of 0.2 ml min−1. The elution buffer consisted of 20-mM Na HEPES, pH 7.2, NaCl 200 mM and 2-mM DTT. SAXS data were collected continuously, with a frame duration of 1.0 s and a dead time between frames of 0.5 s. Selected frames corresponding to the main elution peak were averaged using FOXTROT, a dedicated home-made application. A large number of frames were collected during the first minutes of the elution, and these were averaged to account for buffer scattering, which was subsequently subtracted from the signal during elution of the protein. Data reduction to absolute units, frame averaging and subtraction were done using FOXTROT. All subsequent data processing, analysis and modeling steps were carried out with PRIMUS and other programs of the ATSAS suite (http://www.embl-hamburg.de/biosaxs/atsas-online/).

Shapes of the M1–HP4 complex with the C-terminal domain were restored from the experimental data using the program GASBOR (55). These were averaged to determine common structural features and to select the most typical shapes using the programs DAMAVER suite.

The best model among those created for the RNA (M1-HP4) with P65 manually docked on the apical loop was sorted out by fitting with CRYSOL to the SAXS experimental data. The position of the P65 on HP4 was then further refined with program SASREF by rigid body molecular modeling against the shapes of the complex calculated from the SAXS data (56). In this last step of the modeling process, the nucleotide G312 interaction with the C-terminal domain was considered as a supplementary distance constraint.

## RESULTS

### Defining the domains of LARP7 required for the study

The sequence of LARP7 (582 amino acids, in human) comprises three regions (Figure 1B). Following a short unfolded region containing positively charged amino acids (1–27), the La module comprises two structured domains, one containing the LAM (residues 28–111) and the second, an RNA-recognition motif (RRM1; residues 120–199) according to a global analysis of the LARP superfamily (1). At the C-terminus, a domain comprising RRM2 (residues 450–545) has been hypothesized to be similar to the xRRM domain found in P65, a protein involved in the telomerase complex in *Tetrahymena* (50). The xRRM fold differs from most RRMs because of the peculiar folding property of its C-terminal helix, which dramatically extends when binding to its RNA target (54). Between these folded N- and C-terminal domains, most of the central region of LARP7 is predicted to be unfolded, except a short region around residue 400 just before RRM2 (Figure 1B). In addition, the extreme N-terminus and the linker region contain stretches of basic residues.

In the prospect of elucidating how these modules are combined in LARP7 to bind specifically the 7SK RNA, we expressed in *E. coli* the full-length LARP7, the La module comprising the LAM and RRM1 regions with an N-terminal extension (1–208) and the C-terminal domain comprising the RRM2 (433–582). Several boundaries were tried for the La module, but only 1–208 was considered for a structural analysis (see details in the Supplementary Material section). Elucidation of the role of the C-terminal RRM2 was approached by biochemical experiments with the construct (433–582). Crystallization assays were focused on complexes of the La module with RNA.

### Crystallization of a complex of the LARP7 La module with RNA

Binding to RNA was monitored *in vitro* by EMSA experiments (Figure 2). These showed that 7SK RNA and LARP7 interact without any additional partner and confirmed that 7SK truncation of the 3′ polyU reduces the binding of LARP7 about 1.6 times (Figure 2A), as anticipated from the homology with La (3). Further truncation of the entire 3′-hairpin in 7SK-ΔHP4 (1–295) led to further loss of binding (Figure 2A), reducing affinity about 2.2 times. Interestingly, this indicated that other parts of 7SK are involved in binding the full-length LARP7. The present study was mainly focused on the 3′-end domain of 7SK comprising the HP4 hairpin (300–331; Figure 1A), which is predicted in all 2D models of 7SK and is the only subdomain for which a 3D structure (PDB 2KX8) is available (53). With the La module (1–208) and the RNA corresponding to the 3′-domain, the truncation of the 3′-end uridines showed a drastic effect (Figure 2B), thus confirming that the 3′-uridines of 7SK are essential for the La module binding.

The crystallization assays included single-stranded oligonucleotides (325–332, 314–332) as well as RNAs comprising the HP4 hairpin (302–332, 300–332, 287–332). Thermofluor experiments (57) showed a considerable increase of protein stability, with a *T*_m_ change from 26° to 43° upon RNA binding. Therefore, RNAs were mixed with purified protein prior to concentration and set-up of crystallization trials. Similar crystals were obtained in similar conditions with all RNAs, but very few of them diffracted well. The structure was solved from a crystal obtained in a drop initially set up with the hairpin HP4 (300–332). However, a check of the drop content after crystal mounting showed that the RNA was degraded. The formation of crystals with similar unit cells in drops initially containing RNAs which all comprised the 325–332 sequence immediately suggested that this short UUUCUUUU-3′ stretch of 7SK sequence was the longest oligonucleotide possibly present in the crystal. Indeed, difference Fourier showed densities for only three and five nucleotides in monomers A and B, respectively (Supplementary Figure S1). The electronic densities corresponded to pyrimidines and were interpreted as 5′-CUUUU-3′.

### Global view of the structure of the La module of LARP7

The asymmetric unit of the monoclinic crystals contains two protein molecules. The best defined monomer B will be described in the following text (Figure 3A). Although they share only 34% sequence identity, the La module of LARP7 showed a great degree of structural similarity with the La module of HsLa (6), as indicated by an RMSD of 2.0 Å for 188 residues. Figure 3A shows the two subdomains, LAM and RRM1 with the characteristic architectures observed previously in HsLa (5–6,58–59). Namely, the topology of the LAM subdomain is that of a winged helix-turn-helix, a fold often encountered in transcription factors involved in DNA binding, but with helices α2 and α3 inserted into the standard winged helix-turn-helix. It comprises thus six helices and two short β-strands. The N-terminal residues (1–28) are not visible in the map.

RRM1 shows a variant form of the RNA recognition fold found in many RNA-binding proteins (4), an ancient and abundant fold built around a central β-sheet, with two helices packing against one face (Figure 3A and B). Most RRMs use the central β-sheet surface to bind RNA. This surface is characterized by a cluster of aromatic residues, from hallmark sequences RNP1 and RNP2, located in β3 and β1 strands, respectively (Figure 3B and Supplementary Figure S2). As expected from its early identification as an RRM (60), the 3D structure of the La protein showed the RRM1 adjacent to LAM to be standard (5–6,58–59). Most LARP proteins contain RRM-like variants, as for example LARP6 (7). Interestingly, the present crystal structure of the human LARP7 shows RRM1 to be smaller than the standard fold, with a β-sheet of only three strands (Figure 3B and C). Although strand β4 is missing, the essential part of the β-sheet is maintained. The aromatic residues of the motif signatures, here Tyr128 from the RNP2 and Phe170 from the RNP1, are solvent-exposed (Figure 3B). Loop 3 connecting strands β2 and β3 is quite long (Figure 3A). It comprises two groups of two residues (158–159 and 164–165) facing each other, and forming two very short strands according to the secondary structure determination program STRIDE (61). Helices RRM-α1 (138–147) and RRM-α2 (176–184) pack against the other face of the β-sheet (Figure 3A). There is no additional C-terminal helix (α3 in HsLa), but helix α2 is one turn longer than in La, and extends to the last visible residue, Asn188. To rule out the possibility that our design of the protein was too short to include helix α3, we attempted to produce a larger protein construct encompassing residues 1–228, but unfortunately it was poorly soluble and could not be used in crystallization or binding studies. On the linker side, there is an N-terminal Helix RRM-α0 (121–125), as in La, but much shorter, and reduced to one helical turn. Together, these differences contrive to make the RRM1 domain of LARP7 singularly small. This is highlighted in Figure 3C, where the structures of the La modules of LARP7 and La were superimposed.

In LARP7, the linker connecting RRM1 to LAM follows a similar path as in HsLa, and has a similar size of nine residues (10 in HsLa) between the RR sequence from the ‘wing 2’ motif (Arg110-Arg111), which marks the C-terminal boundary of the LAM domain (7) and the first residue (Asp121) from the RRM-α0 helix. Interestingly, the connection between LAM and RRM has recently been hypothesized to impact the relative orientation of the LAM and RRM1 domains, and thus the RNA-binding property of the protein (7). In LaRP7 the path of the linker is constrained by a salt bridge, between Arg118 and Glu122 and the relative orientation of the domains is maintained by a conserved salt bridge between Lys53 and Glu172 (Figure 3B). This results in an orientation similar as in HsLa. Together, the LARP7 La module composes a stable structure closing on the 3′-end of the RNA.

### Specific features of the RRM1 of LARP7

The larger stretches of residues absent from the sequence and structure of LARP7 are highlighted in blue in the 3D structure of La (Figure 3C). The largest stretch corresponds to the missing fourth β-strand and the C-terminal helix α3 of the RRM1. Another missing stretch corresponds to the amputation of helix RRM-α0. Interestingly, these deletions are clustered in 3D and align continuously along the same surface, opposite to the uridine-binding cleft (Figure 3C). Together, these deletions suggest that RRM1 in LARP7 could have special properties. This is supported by the multiple sequence alignment shown in Supplementary Figure S2 where LARP7 and La sequences were compared. Several residues of the β-sheet are conserved only in LARP7, and not in La sequences. One of these is Glu130, adjacent to the Tyr128 of the RNP2, at the edge of the β-sheet opposite to the binding site of the 3′-uridine (which is described in the next section). Interestingly, Phe168, a hallmark residue of the RNP1 in RRMs (4), present in LARP7, is a serine in La. On the other face, the two helices are packed closer to the β-sheet in the LARP7 structure. The contact involves several hydrophobic amino acids, as for example Trp140, Phe145 and Phe185. An interesting swapping of residues, conserved in evolution, is observed with Phe145 (Supplementary Figure S2). This phenylalanine comes from helix α1 in LARP7 but from helix α2 in La.

Taken together, these observations support the 3-stranded RRM as a genuine characteristic of LARP7 proteins. The La module comprising this shortened RRM1 binds the 3′ domain of 7SK (Figure 2B). The existence of a fourth β-strand and α3 helix of the RRM cannot however be totally ruled out in full-length LARP7, as a deep analysis of the alignment showed a weak sequence similarity of the α3 helix with a remote part of LARP7 (amino acids 375–390; Supplementary Figure S2B). If a 4-stranded RRM is formed in LARP7 proteins, it would thus involve a huge insertion of ∼200 amino acids. This still makes the RRM1 in LARP7 very different from classical RRMs (62). Interestingly, LARP6 shows a very different situation, with additional helices blocking access to the RNA-binding face of the RRM (7). Here, the smaller RRM1 domain of LARP7 rather suggests an increased accessibility.

### Recognition of the RNA 3′-terminal triplet

**Figure 4.**
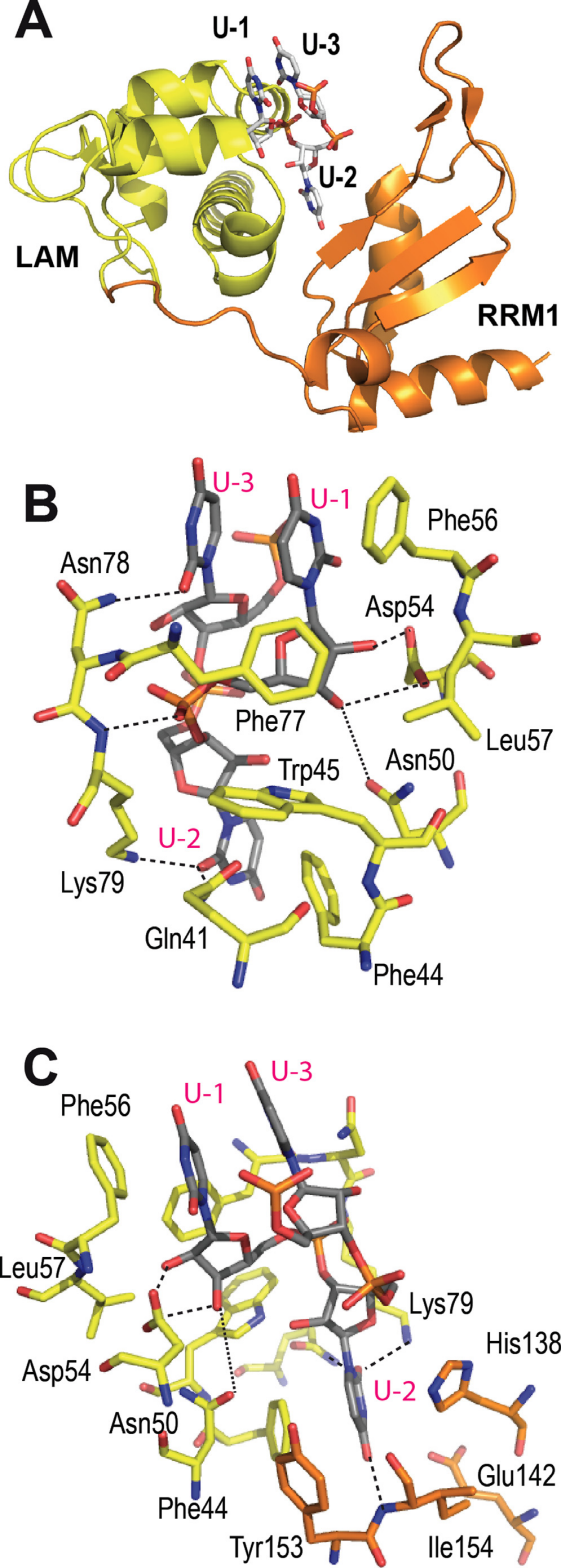
Binding site of the terminal uridines in LARP7. (**A**) LARP7 La module with LAM domain in yellow and RRM domain in orange, showing the arrangement of the terminal uridines. (**B**) Details of the 3′ uridines binding site viewed from the LAM side, showing the terminal ribose binding Asn50 and Asp54 and the stacking of U-1 and U-3 with Phe56, in stick representation, with carbons gray for RNA, yellow for amino acids from LAM. Dotted lines indicate H-bonds (distances in the range of 3.0–4.0 Å). (**C**) Perpendicular view, turned around the vertical axis, showing the specific recognition of U-2 by the RRM1, in orange.

In the electronic map, three uridines in one monomer and five nucleotides in the second monomer are visible in the cleft formed between LAM and RRM domains (Figure 4A and Supplementary Figure S1). The three terminal uridines from the two monomers superpose well, and the following description depicts the monomer showing five residues. Most interactions involve H-bonds with residues of the LAM domain (Figure 4A and B). As was observed in La protein (6), the penultimate U-2 (numbering as in La) is anchored at the bottom of the crevice, and U-1 (the 3′-terminal uridine) at the surface of the LAM domain. The uridine U-3 stacks on U-1, leading to a characteristic fishhook shape of the backbone (Figure 4A).

The base of the terminal uridine U-1 stacks on Phe56 from the LAM-α3 helix. The stacking of the base of U-3 further restricts the binding pocket, which is limited at the bottom by Phe77, lying on the sugar ring. The terminal ribose binds Asn50 and Asp54 (Figure 4B). Both 2′ and 3′ hydroxyl groups from U-1 are bound simultaneously, thus ensuring that the ribose is 3′-terminal. All residues involved in U-1 binding are invariants in LARP7 and La (noted $ in the alignment; Supplementary Figure S2), including Asn50, which was not reported as binding the RNA in HsLa. The distance observed with the 3′-OH in LARP7 is quite long (3.9 Å), but still compatible with H-bond formation, and shorter than the distance in HsLa (4.4 Å). Interestingly, this binding pocket, open to the solvent at the base edge, is not specific for uridine. In the course of our study, the binding of LARP7 N-terminal domain was measured with RNA variants of the terminal residue. The rationale was to test whether LARP7 could distinguish mature 7SK (331 nucleotides ending by CUUU) from transcript (332 residues, CUUUU) or maturation intermediates, such as a version with 332 nucleotides and a terminal adenine (CUUUA) that was mentioned in an earlier study (63). No binding differences were observed in EMSA experiments with these variants (Supplementary Figure S3). Similar absence of discrimination was observed with the full-length 7SK of 331 or 332 nucleotides. However, when the RNA was produced by self-cleavage from a transcript containing a 3′ ribozyme (which was attempted to produce homogeneous molecules with defined 3′-terminal ends to improve homogeneity for crystallization (64)), the binding efficiency was decreased. This was ascribed to the presence of a 2′-3′ cyclic phosphate resulting from the cleavage by the ribozyme. This highlights that the 2′ and 3′ hydroxyls of the terminal ribose must be free for LARP7 to bind, while the nature of the terminal base is of less importance. Similar results showing that the sequence of the 3′-end residue was not essential were obtained with HsLa (59).

Specific binding of the penultimate residue U-2 involves residues from both LAM and RRM1 domains (Figure 4C). The base ring is sandwiched between Phe44 from LAM and His138 from RRM1, in a pocket closed by Tyr153 from RRM1. Interestingly, in La, the bottom of the U-2 binding pocket is also closed by a tyrosine (Tyr23 in human La), which comes from the LAM side. Specific binding of the pyrimidine ring O2 atom results from H-bonds with Gln41 and Lys79. The O4 atom characteristic of uridine faces the RRM domain and points toward the β-sheet, between the β2 edge and the RRM-α1 helix (Figure 4C). This arrangement provides for one H-bond with the main chain nitrogen of Ile154. Residues His138 and Glu142, from the RRM-α1 helix are in correct orientation for H-bond formation; however, in the present crystal, the distances are slightly too long (above 4 Å). Most of the residues participating in the U-2 binding site belong to a group of residues specific of LARP7 proteins, as revealed in the sequence alignment (Supplementary Figure S2). Among those, His138 is 89% conserved. Ile154 is 50% conserved and can be replaced by a valine (50%). The others (Trp140, Phe145, Tyr153, Ser155, Pro157) show conservation above 94%. On the LAM side, Asn78 is 94% conserved and Lys79, 82% conserved, may be replaced by an arginine. Besides its stacking on U-1, U-3 is bound by Asn78 at its O2 atom, but is not further stabilized at the O4 edge, which faces the solvent. The same situation was observed in HsLa (6).

### Potential binding to other parts of the RNA

The upstream nucleotides, U-4 and C-5, are only visible in one monomer of the asymmetric unit. They lie approximately in the same planes as U-3 and U-1, respectively, as shown in Supplementary Figure S4A. The ribose-phosphate chain is driven apart from helicity, with the bases U-4 and C-5 unstacked. This arrangement could be linked to the proximity of the long loop 3 connecting β2 and β3, which was previously involved in RNA binding (4). In LARP7, loop 3 can be pictured as a guide pushing the RNA on the LAM surface into the binding cleft, as highlighted in Supplementary Figure S4A. In that context, Lys160, which here stacks on the U-4 base, could play a prominent role. Indeed, together with a Tyr159, this residue is quite conserved in LARP7 sequences (Supplementary Figure S2).

In the crystal, nucleotides U-4 and C-5 are involved in a packing contact involving the β-sheet of a neighboring molecule (Supplementary Figure S4A). The RNA is facing the aromatic residues from the signature sequences RNP1 (Phe168 and Phe170) and RNP2 (Tyr128). This suggests that the β-sheet in LARP7 RRM1 may bind RNA. Analysis of the packing contact does not reveal direct interactions with the RNA, apart from Tyr128 stacking on U-4. Interestingly, the mutation of Tyr128, hallmark of the RNP2, was reported in an earlier work to result in a loss of binding specificity to 7SK (65). Residue Glu130, one of the residues specific of LARP7s observed in the sequence alignment (Supplementary Figure S2), 94% conserved, is found in the vicinity of C-5. It is positioned near the nucleotidic base, suggesting that it could participate with Tyr128 to 7SK recognition. Phe168, a hallmark of RNP1, is stacking on Tyr128. To further clarify their role in RNA binding, residues Glu130 and Phe168 were mutated to alanines. We observed that RNA binding was not affected for the F168A mutant, but decreased for the E130A mutant (Supplementary Figure S4B). Checking by circular dichroïsm indicated that the E130A mutant protein showed the same global conformation as the wild-type. This suggests that the LARP7-specific residue Glu130 at the β-sheet edge of RRM1 is involved in 7SK binding.

### Binding of the C-terminal RRM2 to 7SK

Most RRM-containing proteins have at least two RRMs, which often combine, leading to an expanding wealth of RNA- and protein-binding catalog. We therefore set out to investigate the function of the second, RRM2 domain at the C-terminus of LARP7.

**Figure 5.**
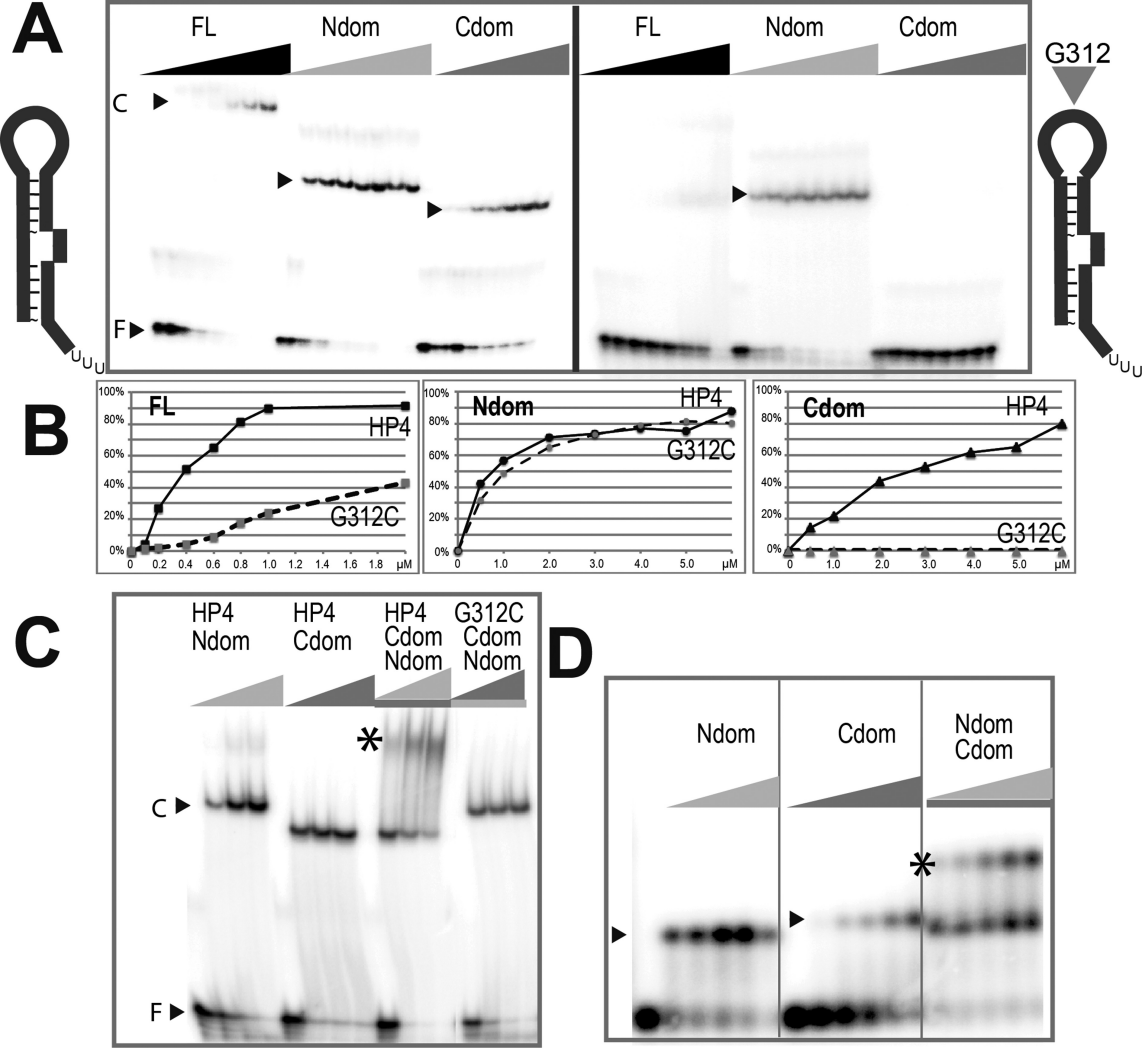
The C-terminal domain of LARP7 binds the apical loop of the 3′ hairpin of 7SK. (**A**) EMSA comparing binding of wild-type RNA HP4 (left panel) and mutant G312C (right panel) with LARP7 full-length (FL), La module (Ndom) or C-terminal domain (Cdom). (**B**) For each protein, the complex formation with wild-type or mutant HP4 reported as a function of protein concentration. (**C**) EMSAs of HP4 incubated with La module- (Ndom), C-terminal domain (Cdom) or both domains or mutant G312C (HP4mut) incubated with both domains. The supershift is labeled with a star (*). (**D**) Binding of the 5′-extended 262-HP4 RNA with the N- (Ndom), C- (Cdom) or both domains, analyzed with a native agarose gel. The supershift is labeled with (*).

A construct encompassing the C-terminal RRM2 (433–582) was assayed in binding experiments. It showed strong binding to several RNAs derived from 7SK, including those restricted to the 3′-hairpin, HP4 (Figure 5A and B). A recent breakthrough into specific recognition came from a mutational analysis establishing that position G312 in the apical loop of hairpin HP4 is essential for the 7SK to be correctly bound *in vivo* (32). In agreement with the *in vivo* experiment, EMSA assays *in vitro* with purified full-length LARP7 showed that the mutation G312C strongly reduced the binding with HP4. This mutation also abolished the binding with the RRM2-containing domain (Figure 5A and B), thus showing that this domain binds to the apical loop of HP4. Mutation G312C did not, however, decrease the binding of LARP7 N-terminal domain, indicating that it does not contact the G312 position. Considering that the La module binds the 3′-end uridines, this suggests that LARP7 folds back to position its C-terminal domain on the terminal hairpin of 7SK. Indeed, it was possible to bind simultaneously the N- and C-terminal domains of LARP7 on RNA constructs containing the 3′-hairpin of 7SK, and observe supershifted bands (Figure 5C and D), regardless of the order of addition of the two proteins.

**Figure 6.**
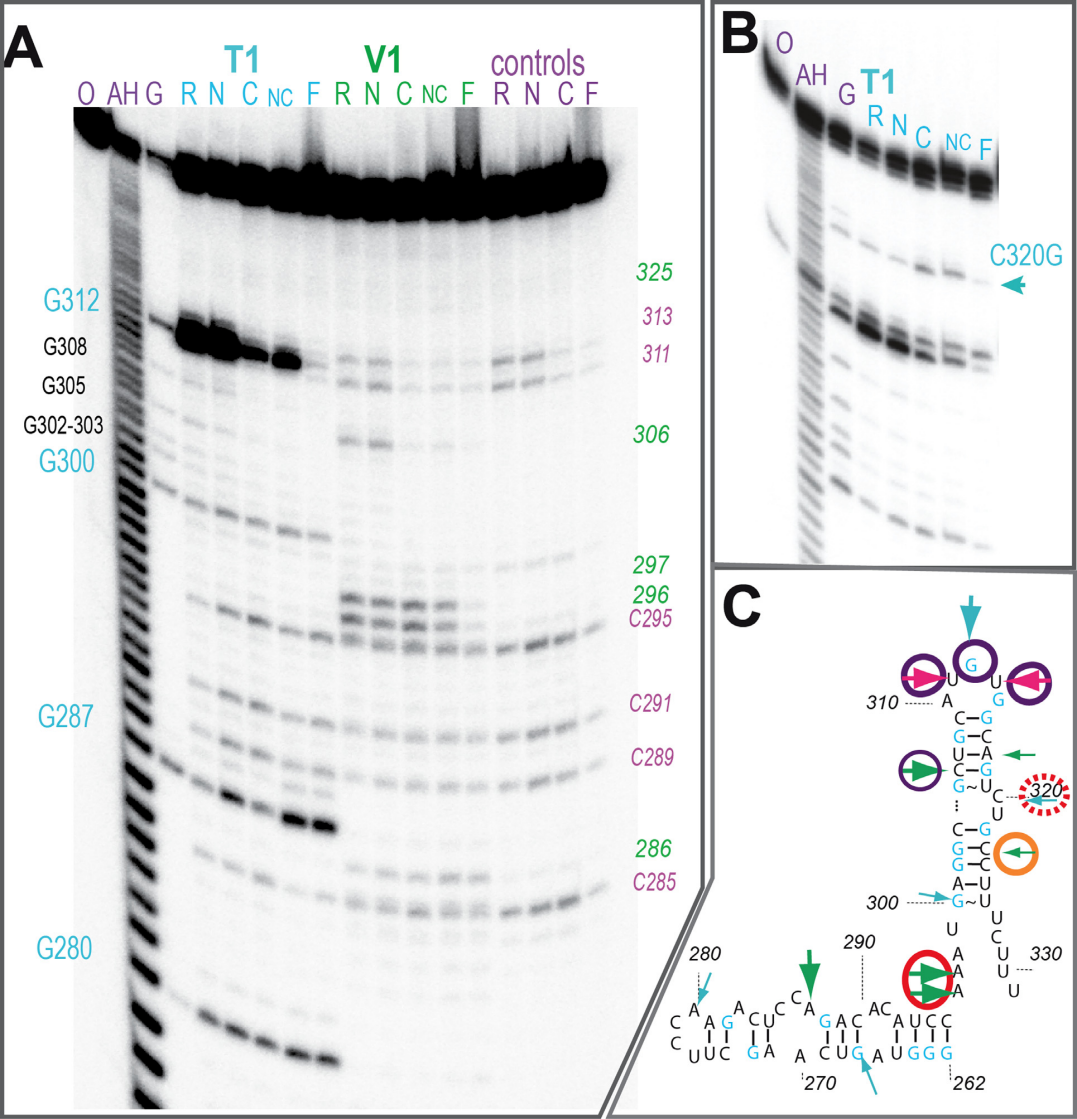
Localization of the binding sites of the N- and C-terminal domains on the 3′ hairpin of 7SK. (**A**) Footprinting experiment of the 5′-labeled 262-HP4 RNA with RNases T1 and V1. Denaturing gel showing the cleavage products of the free (R) and complexed RNA with domains N- (N), C- (C), both (NC) or full-length LARP7 (F). Sequence was indexed with T1 in denaturing conditions (G) and ladder (AH); cleavages positions are indicated in green (V1) or blue (T1); control without treatment (C) shows in-line (purple) cleavages. (**B**) The same experiment but with the mutated 262-HP4 C320G. (**C**) Summary of the footprinting results. Arrows show cleavages with RNAse T1 (blue), V1 (green) or in-line (pink). Circles represent protections with domains N- (orange) and C-terminal (purple) or full-length LARP7 (or red).

Knowing the tendency of RRM domains to pack together, supported by packing contacts observed in the crystals of LARP7, we wondered whether an interaction could be established between RRM1 and RRM2. However, binding the La module to the 3′-end of a hairpin mutated at G312 did not recruit the RRM2 domain (Figure 6C). Simultaneous binding of the N- and C-terminal domains on the 3′-hairpin thus seems driven by RNA and not by interactions between the domains. More data will be required to investigate the situation when the domains are linked by the central region of LARP7.

### Footprinting investigation of the positions of LARP7 N- and C-terminal domains on the 3′ hairpin

To position the N- and C-terminal domains on the 7SK 3′-hairpin, we used footprinting experiments (66) and compared the accessibility of nucleotides to RNases in free RNA and RNA complexed with the three protein constructs (Figure 6). The RNA was 262-HP4 (262–331; Figure 1A). We used RNase T1, which recognizes the guanosine base when it faces the solvent and cleaves the ribose-phosphate chain on the 3′ side of guanines, and RNase V1, which cleaves structured regions. In the absence of protein (Figure 6A, lanes R), RNase T1 strongly cleaves the hairpin loop at G312, while V1 cleaves in the helical regions of the hairpin on both sides of the loop. LARP7 C-terminal domain protects G312 from T1 cleavage, as well as the adjacent nucleotides 311 and 313 from in-line cleavage. The protection extends to the 5′-side of the loop, to the V1 cleavage at C306. The N-terminal domain protects a weak V1 cleavage at 325, in the stem just on top of the 3′-terminal single-stranded tail.

At the foot of the HP4 hairpin, the adenines A296–A297 show strong V1 cleavages, indicative of base-pair formation. They might possibly pair with the terminal poly-U, but the V1 signal is unchanged upon complexation with the N-terminal domain, which captures the 3′-end. This suggests that A296–A297 connect elsewhere in 7SK. Binding of full-length LARP7 induces the disappearance of this V1 cleavage, which suggests a protection induced by the central linker region. An alternative explanation is that the structure probed by the V1 cleavage melts upon LARP7 binding, suggesting a conformational response of the RNA upon LARP7 binding, a situation reminiscent of the telomerase case (54). This hypothesis requires further investigation.

**Figure 7.**
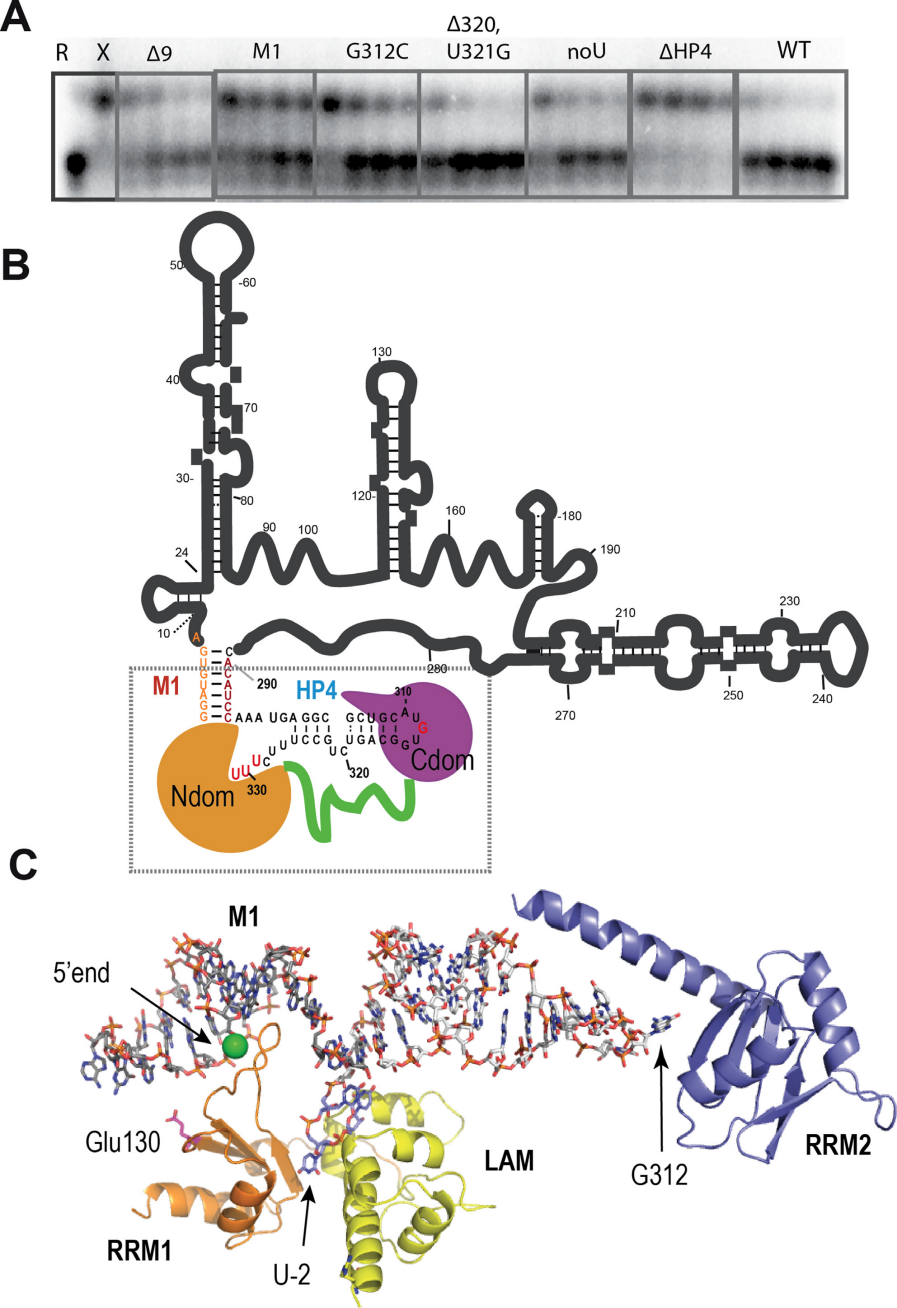
Model for the assembly of LARP7 N- and C-terminal domains on the 3′ hairpin of 7SK. (**A**) Impact of 7SK mutations on the binding with LARP7, estimated by competition experiments. R and X show the migration of the P^32^-labeled RNA when 7SK is free (R) or bound to full-length LARP7 (X). Increasing amounts (250, 500, 750 or 1000 nM) of mutant RNAs were incubated together with 7SK (50 nM) before adding LARP7. RNAs were 7SK full-length (WT), 1–295 (ΔHP4), 1–328 (noU), mutated at the HP4 bulge (Δ320, U321G) or apical loop (G312C) or at the sequence 289–295 (M1) or deleted of residues 1–8 (Δ9). (**B**) 2D model showing the M1 region, connecting the 5′-end of 7SK (orange) with the 290–295 sequence (red) and the binding positions of the LARP7 domains N- (orange) and C- (purple) with the linker in green. (**C**) Our working model, corresponding to the squared region in (B), showing the M1-HP4 RNA with the N-terminal domains (LAM, orange and RRM1, yellow) and the C-terminal domain, represented by the structure of P65 (PDB id. 4ERD, purple). The 5′-terminal phosphate of 7SK is indicated with a green sphere.

Before using the footprinting information to guide docking experiments, more information was required regarding the bulge at 320–321. The presence of a bulge at 320–321 in the upper part of hairpin HP4 was found to be essential for LARP7 binding in the *in vivo* study (32), but its sequence seemed to be free, as it could be changed without impacting LARP7 binding. Indeed, we checked that changing the bulge, even by a drastic reduction to one residue, did not compromise the binding to 7SK (Figure 7A). We took advantage of this freedom to change C320 for G320, and monitored the accessibility of this guanine with RNase T1. Footprinting of 262-HP4 with the mutation C320G (Figure 6B) showed profiles similar to the wild-type situation, but for an additional weak cleavage corresponding to C320G in the free RNA, a clue that this residue is bulged out as expected. Neither LARP7 domain protected that position. Thus, in LARP7, the RRM2-containing domain seems to bind only to the apical region. Interestingly, this enhanced the T1 cleavage, suggesting an effect on the helicity. The full-length protein (not the combination of N- and C-domains) showed a clear protection of residue 320. This suggests that the central linker region of LARP7 may be involved in RNA binding.

### LARP7 recognition at the foot of the 3′-hairpin supports a closed secondary structure of the 7SK RNA

Interestingly, the 5′-extension in the 262-HP4 RNA is not fully flexible, as indicated by the V1 cleavage at position 286, suggesting the formation of a structure. Indeed, Mfold (67) predicts this extension to form an additional hairpin, represented in Figure 6C. Moreover, we observed that assembly of N- and C-terminal domains was facilitated with 5′-extended constructs of HP4. For example, the RNA 262-HP4 (262–331) showed clear binding with each domain as well as clear supershifts (Figure 5D). Comparison of binding with shorter and longer constructs, as reported in Supplementary Figure S5, shows an increase of affinity for the 5′-extended RNA of ∼25 times for the N-terminal domain, while it does not vary for the C-terminal domain. This suggests that the N-domain binds not only the 3′-end but also the 5′ foot of HP4.

The sequence upstream of HP4 (289–295) is well conserved (12). It has co-evolved together with seven nucleotides at the 5′-end of 7SK, with which it was hypothesized to form seven base pairs, resulting in the formation of a stem (M1; Figure 1, inset). This closes the 7SK in the form of a lariat (37). To further investigate if the M1 region is involved in LARP7 binding, we mutated it in two ways. The M1 mutant was obtained by changing the 289–295 CACAUCC sequence to its complement GUGUAGG, the Δ9 mutant by deletion of the 5′-end (7SKΔ9 starting at G9). Both abolish the formation of base pairs. Binding to LARP7 was monitored by a competition assay (Figure 7A). Both mutations decreased the ability of 7SK to bind LARP7. The M1 mutant showed a strong effect. The Δ9 mutant was weaker but still affected the binding at a level comparable to the deletion of the terminal uridines (Figure 7A). This indicates that the closed fold of 7SK is not only valid but also important for LARP7 binding.

### SAXS study of the complex of LARP7 RRM2 with the 3′ region of 7SK

An RNA named M1-HP4 was designed by linking the 5′-sequence of 7SK (GGAUGUG) to the 3′ region at C299 by a GAAA sequence, and produced by *in vitro* transcription. Modeling with MC-fold (52) indicated that this RNA forms a structure with two hairpins, where the seven base-paired M1 extension closed by a GAAA tetraloop is appended to the hairpin HP4. M1-HP4 was bound efficiently by LARP7. The M1-HP4 sample was mixed with LARP7 C-terminal domain, and the complex submitted to SAXS analysis (Supplementary Figure S6). Starting models of the complex of M1-HP4 with RRM2 were generated by manually docking the structure of P65 on M1-HP4 RNA models obtained with MC-fold (52). This was based on the structural analysis of P65, which was suggested to have a similar structure as LARP7 C-terminal domain (50,54). It was immediately clear that the SAXS experimental curve was best fitted when the P65 was docked on the apical loop. Fitting with the SAXS experimental curve was then used to choose the best among the models provided by MC-Fold. The best model corresponded to a coaxial stacking of the HP4 and M1 hairpins, a favorite in RNA structures. In parallel, the shape of the complex shown in Supplementary Figure S6B was restored from the experimental data using the program GASBOR (55). Finally, starting with the best model for M1-HP4 with P65 grossly positioned at the apical loop, the position of the RRM2 (here P65) was refined by rigid body molecular modeling against SAXS data with SASREF (56). During this last stage of the process, a distance constraint was introduced, to maintain the interaction of nucleotide G312 with the C-domain. This process led to an excellent fit (chi^2^ 2.1) as shown in Supplementary Figure S6A.

### Model of LARP7 domains on the 3′ region of 7SK

The SAXS study thus confirmed that LARP7 C-terminal domain binds to the apical loop of HP4. Moreover, the atomic models generated with the SAXS study allowed a mutational analysis. Based on the alignment of P65 with LARP7 (Supplementary Figure S7A), we chose two residues close to the RNA in the model and conserved in LARP7, but different in P65 and not from the RNP sequences. Residues Tyr513 and Lys517 of P65 align with Lys535 and Asp539 of LARP7, respectively (Supplementary Figure S7A and B). In an EMSA experiment with M1-HP4, we observed that while D539A had no visible effect, the mutation K535A clearly decreased the binding to RNA (Supplementary Figure S7C).

The N-terminal domain was docked manually on the M1-HP4 model, by anchoring the U-4 nucleotide observed in the crystal on C328 from the model. The terminal U triplet was from the structure. This still leaves the N-terminal domain quite free to rotate around the connection. A more precise all-atoms modeling was not attempted, as it will require more data to orient the structural elements with confidence. Interestingly, the residue Glu130 which was suggested to be involved in 7SK-binding (Supplementary Figure S4) is positioned toward the M1 region of the RNA in the working model shown in Figure 7C.

## DISCUSSION

The crystal structure of the LARP7 N-terminal domain, described here, is the first 3D structure of the La module of a member of the LARP family different from La showing the linked domains of the La module in a complex with RNA. Until the recent publication of the individual structures of the two domains of the La module from LARP6 (7), structures were available only for short fragments (68).

The triplet of uridines at the 3′-end of 7SK binds into the cleft between the LAM and RRM1 domains. It is constrained in a characteristic hooked conformation, allowing strict recognition of the penultimate uridine, with a contribution of the RRM1 revealing LARP7 specificity. The relative orientation of the two domains of the La module seems to be, as in HsLa essentially driven by the 3′-terminal uridines binding in the cleft between the domains, with U-2 located exactly at the same position. In the course of the La structural analysis, it was hypothesized that the two domains move freely in the absence of RNA (6), a hypothesis developed in the recent structural analysis of LARP6 that suggested a participation of the sequence at the exit of the LAM domain to the topological arrangements of the LAM and RRM1 domains (7). This movement may be restricted in LARP7, where a conserved salt bridge (Lys53 with Glu172) impacts the relative positions of the domains.

Comparing the present structure with HsLa shows that while the LAM domains are similar, the RRM1 are different. In LARP7, a smaller RRM1 is formed, with its β-sheet composed of three instead of four strands. The absence of the C-terminal α3 helix of RRM1 combines with the reduction of the length of the N-terminal helix of the RRM to increase the accessibility to the RNA-binding residues of the central β-sheet of RRM1. In RRMs, the fourth β-strand often contributes H-bonds for the specific recognition of the RNA substrate. In LARP7, while it cannot be excluded that a fourth β-strand and α3 helix are recruited from downstream sequences, possibly via the RNA, the RRM1 shows several specific residues that could play a role in binding RNA. Residues including His138 and Ile154 were seen to participate in the penultimate uridine recognition, in a different way compared to La, thus showing a LARP7-specific response to a common task. Another residue, Glu130, also identified as LARP7-specific in the sequence alignment was shown by mutation to be involved in binding the 3′-terminal domain of 7SK. Glu130 is on the other side of the β-sheet, too far to be involved in the recognition of the terminal uridines. It may be participating in the specific function of LARP7, which is the recognition of 7SK. Further work will be necessary to identify the eventual binding site on 7SK. Interestingly, the RRM1 is very different in LARP6, where the RNA-binding surface is blocked by additional helices (7).

The 3′-hairpin of 7SK, HP4, was recently elegantly demonstrated to be the specific target of LARP7 *in vivo* (69). We now show that recognition of the 3′-end of 7SK occurs jointly through the N- and C-terminal regions of LARP7. Binding experiments and footprinting revealed that the C-terminal domain binds the apical loop of the HP4 hairpin. This domain is homologous to the telomerase protein P65, which forms an xRRM fold, an RRM with an extended C-terminal helix. In the telomerase, the RNA recognition depends on a two-nucleotide bulge, which is located in the middle of the hairpin. In 7SK, the HP4 hairpin also has a bulge, which was previously shown to be required for LARP7 binding *in vivo* (32), but without sequence specificity. The bulge in 7SK is not recognized by RRM2, but may be necessary to facilitate the packaging of 7SK into a functional conformation.

The N-terminal domain binds not only the terminal uridines but also the 5′ region at the foot of the 7SK 3′-hairpin. This sequence, which is highly conserved, has been previously hypothesized to form seven base pairs with the 5′-end of 7SK, thus forming a small stem named M1 (37). We show that mutations destabilizing this stem compromise LARP7 binding, thus giving experimental evidence that 7SK is closed in the form of a lariat. Binding of the La module to the M1 stem, 5′ of the HP4 hairpin also explains why increasing the distance between HP4 and the 3′ uridine triplet compromises the binding of LARP7 *in vivo* (32).

The 7SK RNA is 5′-capped by the methyl-transferase MePCE (10), which has been shown to remain bound to 7SK after performing methylation (29). Interestingly, it was shown to bind LARP7 in that process (29). A closed 7SK, where the 5′ MePCE binding site is close to the 3′ LARP7 binding site, clearly facilitates this interaction. In our 3D model, accordingly, the 5′-end of 7SK (represented by a green sphere in Figure 7C) is free to bind MePCE.

We show that two domains of LARP7 bind 7SK, in a head-to-tail arrangement schematized in Figure 7B. The middle region of LARP7, which comprises stretches of basic residues, may also participate in the binding. This is suggested by the observation that full-length LARP7, but not the combined domains, induces protections from RNase cleavage at two positions in HP4, the bulged C320 and the A296–A297 at the foot of the hairpin. Additional binding to the 7SK core outside of HP4 is not excluded either. This is indicated by the observation that deletion of the entire 3′-hairpin still allows complex formation.

Several recent reports of major disfunction in humans suggested that LARP7 and 7SK work as a pair to regulate the transcription factor P-TEFb. The present work showing how LARP7 is entwined with the 7SK RNA suggests functional correlations. Firstly, LARP7 binding could narrow the range of 7SK conformations, thus facilitating recognition by stabilizing a functional RNA structure. Secondly, LARP7 binding may help compact the RNA, by minimizing the phosphate–phosphate repulsions with its basic stretches of residues working as polyamines in the packaging of nucleic acids in viral capsids. Such a chaperoning mechanism could be aided by MePCE binding to the 5′-end. Thirdly, complex formation leads to reduction of the RNA surface accessible to other partners, such as HEXIM, PTEFb or hnRNPs. A heterologous RNA–protein surface, as observed in the Tat-TAR system, was shown to operate in P-TEFb recognition (70). LARP7 binding to HP4 could provide such a surface, in line with an early report showing the importance of HP4 for P-TEFb inactivation (31). The combined effects would increase the specificity of the system. Indeed, as mentioned previously, HEXIM binds RNA with a poor specificity (35,71). In that perspective, LARP7 could, in addition to protecting 7SK from exonucleases, assure the required specificity for 7SK to function in the crowded nucleoplasm of human cells.

## ACCESSION NUMBER

PDB: 4WKR.

## SUPPLEMENTARY DATA

Supplementary Data are available at NAR Online.

SUPPLEMENTARY DATA
