# Supplementary material for: Structural insight into the mechanism of stabilization of the 7SK small nuclear RNA by LARP7
Source: Nucleic Acids Res. 2015 Mar 9;43(6):3373–88. doi: 10.1093/nar/gkv173 (PMC4381077; doi:10.1093/nar/gkv173)
Supplement: SUPPLEMENTARY DATA [file supp_43_6_3373__index.html]

Structural insight into the mechanism of stabilization of the 7SK small nuclear RNA by LARP7 — Structural insight into the mechanism of stabilization of the 7SK small nuclear RNA by LARP7 — SUPPLEMENTARY DATA 

# Structural insight into the mechanism of stabilization of the 7SK small nuclear RNA by LARP7

## SUPPLEMENTARY DATA

**Files in this Data Supplement:**

- SUPPLEMENTARY DATA
