# Supplementary material for: Structural insight into the mechanism of stabilization of the 7SK small nuclear RNA by LARP7
Source: Nucleic Acids Res. 2015 Mar 9;43(6):3373–88. doi: 10.1093/nar/gkv173 (PMC4381077; doi:10.1093/nar/gkv173)
Supplement: SUPPLEMENTARY DATA [file supp_gkv173_nar-03531-m-2014-File010.pdf]

Supplementary material

Supplementary text

Material and methods (supplementary details)

Design of RNAs

The choice of the RNA constructs was based on the sequence of the gene comprising an additional U at position 332. The RNAs assayed for crystallization of a complex with the La module of LARP7 were 302-332, 300-332 and 287-332 comprising the HP4 hairpin, and the oligonucleotides 314-332 and 325-332. While EMSA showed clear binding of the La module of LARP7 with RNAs comprising the HP4 hairpin (Figure 2), it did not with single-stranded oligonucleotides 314-332 or 325-332, suggesting the complex to be too unstable to withstand the electrophoretic process. However, 325-332 (UUUCUUUU) induced stabilization in a thermofluor experiment, where Tm varied from 26°C for the free protein to 43°C for the bound protein. It was thus included in the crystallizations.

Design of proteins

Four proteins encompassing the La module of LARP7 were designed for the structural study, including or not the N-terminal low-complexity region: 1-228, 28-228, 1-208 and 28-208. Only 1-208 could be purified in quantity and quality compatible with crystallization. Similarly, several proteins encompassing the RRM2 of LARP7 were assayed for binding to HP4, among which 433-582 gave the best purification yield.

Preparation of RNAs and proteins.

All RNAs (see below), apart from the 8-mer oligonucleotide UUUCUUUU, synthetic, from Dharmacon, were obtained by *in vitro* transcription of appropriate templates with T7 polymerase and purification of the RNAs on denaturing gels. Templates for transcription were obtained either by annealed oligos or by PCR, starting from plasmids of the type pHDV {Walker, 2003 #395} into which each construct was cloned and primers chosen to produce a transcript ending with U331-3’OH or U332-3’OH. In the case of the RNA starting at 300, A301 was changed for a guanine to facilitate T7 transcription. Crystallization-grade RNAs were

purified by anionic exchange on MonoQ followed by extensive dialysis against storage buffer (10 mM Na cacodylate, pH 6.5, 2 mM MgCl2, 0.25 mM EDTA).

LARP7 full-length and the truncated versions were cloned in bacterial expression plasmids of the pnEA family {Diebold, 2011 #757}, derived from pET15. The plasmid producing an N-terminal His-tag (pnEA-NH) and TEV cleavage site was chosen for all proteins, except for full-length LARP7 which was produced with a C-terminal His-tag, and the crystallized N-terminal domain (amino acids 1-208) for which the P3C cleavage site was used. Expression of proteins, after transformation in *E. coli* BL21-derived strain Rosetta (Novagen), was obtained by culture in auto-inducible medium at 28°C. Lysis was performed by sonication in the presence of 50 mM Tris, pH 7.6, 500 mM NaCl, 5 mM $MgCl_2$, 1.4 mM ß-mercapto-ethanol and protease inhibitors. After debris elimination, purification was performed in batch mode on Ni-beads. The tag was cleaved by overnight incubation with TEV (or P3C) during a dialysis into 20 mM Sodium HEPES, pH 7.2, 1 mM EDTA, 2 mM DTT and NaCl (300 mM for LARP full-length, 200 mM for domains). A cationic exchange chromatography was performed on HiLoad SP-Sepharose (GE Healthcare), followed by dialysis in storage buffer (same as dialysis buffer, above) and concentration by ultra-filtration. For biochemical usage, proteins at 10-50 µM were kept in aliquots at -80°C after addition of 10% glycerol and flash-freezing in liquid nitrogen. For crystallization, the final dialysis step was replaced by a size-exclusion chromatography on Superdex 75.

Circular dichroism

A potential unfolding of the E130A mutant version of the La module was checked by circular dichroïsm (CD). CD spectra were recorded using a Jobin-Yvon Mark VI circular dichrograph at a scan speed of 0,2 nm/s. Quartz spare cuvette with 0,1 cm path length was used. Blanks were run before each spectrum and subtracted from the raw data. Three spectra were averaged to increase the signal-to-noise ratio. The final proteins (wild-type and E130A) were in Hepes buffer (same buffer as in the final step of protein purification) or phosphate buffer (100 mM phospahte pH 7.5) and the assays were carried out at 20°C. The results are presented as normalized $\Delta\varepsilon$ values on the basis of the amino acid residue mass of 110 Da. Taking into account a sensitivity of $\delta(\Delta A) = 10^{-6}$ for the apparatus, the protein concentration and the optical path-length of the cuvette, measurements were obtained at a precision of

$\delta(\Delta\varepsilon)$ = +/- 0,002 $M^{-1}.cm^{-1}$ per amino acid. The mutant E130A showed similar spectra as the wild-type protein in both buffers.

Crystallization of complexes of La module with RNA

Complexes were formed with 1.2:1 molar ratio of RNA to protein. The concentrations were calculated with MW and absorption coefficients from Expasy Website for the proteins, and OligoCalc {Kibbe, 2007 #761} for the RNAs. Complexes were concentrated on Amicon ultrafiltration devices, controlling the concentration following absorption at Λ=260 nm, up to about 5-10 mg/ml. Commercial crystallization kits were tried with a Cartesian robot, at the Structural Biology Platform at IGBMC. Prior to flash-freezing the crystal in liquid nitrogen, the crystals were treated with the cryo protectant, 5% ethylene glycol dissolved in the crystallization solution.

Experimental details for EMSAs and footprinting.

The RNAs were labeled at the 5′-end with T4 polynucleotide kinase (Fermentas) and Y-^{32}P-ATP, following dephosphorylation with antarctic phosphatase (Biolabs). For 7SK, an additional purification on a denaturing gel was performed to eliminate fragments resulting from degradation. Thermal treatment (3 min at 90°C, followed by 5 min on ice) was necessary with 7SK to get one single band on a native gel. This treatment also helped to minimize the formation of duplexes, which formed spontaneously at RNA concentration higher than 0.5 µM, especially in the case of short RNAs such as 302-332. Interestingly, such duplexes elicited efficient binding (Supplementary Fig. S3).

Complexes were formed by incubation at 4°C of 50 nm RNA with proteins in the range of 0-2 µM for full-length and 0-10 µM for domains, in a buffer containing 25 mM Na HEPES, pH 7.2, 5 mM $MgCl_2$, 250 mM NaCl, 2 mM DTT, 0.005% NP40, 10% glycerol, 0.05 mg/ml bovine serum albumin as well as 5 µM total tRNA to minimize non-specific binding. A small amount (1.5 µL) of dye mix containing 0.02% each of bromophenol blue and xylene cyanol in 60% glycerol was added to the 12 µL assay mix just before loading on a native gel in TBE (0.5X). For the small RNAs and hairpins, vertical gels (20 x 20 cm, 1.5 mm thickness) were prepared with 6% acrylamide (29:1), and run at 4 W for 75 minutes. For larger complexes with 7SK, gels (20 x 24, 5 mm thickness) were prepared with agarose (1.6%) and run horizontally in TBE 0.5X at 6W. In this case, RNAs were transferred on nitrocellulose membranes. Gels were

revealed by phosphorimaging. Band intensities were quantified with ImageJ. Curves [bound/(bound+unbound)] were drawn with Excel.

For footprinting, the 262-HP4 RNA was gel-purified after labelling, and incubated at 4°C with or without proteins, in a volume of 10 µL, in a buffer containing 25 mM Na HEPES, pH 7.2, 5 mM $MgCl_2$, 250 mM NaCl, 2 mM DTT, 0.005% NP40, 0.05 mg/ml bovine serum albumin and 5 µM total tRNA. After 15 minutes, allowing for complex formation, 1 µL RNase T1 (Ambion; diluted 1/1000) or V1 (Ambion; diluted 1/10) was added. After 5 minutes cleavage at room temperature, gel-loading buffer was added with either 10 mM EDTA or 10 mM $MnCl_2$ for stopping V1 or T1 reactions, respectively. Reactions were immediately loaded on a sequencing gel (20x40 cm, 0.4 mm thick, 15% acrylamide in urea 8M and TBE), together with an alkaline ladder and RNase T1 in denaturing conditions for sequence indexation, and migrated at 20W.

Supplementary figures legends

Figure S1 Electron density maps at the RNA binding site

Stereoviews of electron density maps at the RNA binding site. (A) Final 2Fo-Fc map generated in the presence of the full model, including RNA, using BUSTER and contoured at 0.8 sigma. (B) Fo-Fc simulated annealing omit map (orange) generated in the absence of RNA using CNS, contoured at 2.5 sigma. (C) 2Fo-Fc simulated annealing omit map generated in the absence of RNA using CNS, contoured at 0.8 sigma.

Figure S2 Multiple sequence alignment of La and LARP7

Multiple sequence alignment of La and LARP7 showing the secondary structures depictions from the LARP7 structure (top) and La structure (2VOD, bottom). **(A)** La module with LAM in yellow, and RRM in orange. Blue arrows indicate the signatures sequences of RNP-1 and RNP-2. The residues binding the uridines triplet in LARPs7 and HsLa are marked by a blue $. Specifically conserved residues are highlighted (blue for LARP7, green for La). Numbers on top correspond to residues number in the human LARP7 sequence, bottom numbers index the columns of the alignment. **(B)** Downstream sequences showing the 4th β-strand and C-terminal helix of the La-RRM1.

Figure S3. Tolerance towards variation at the terminal residue at the RNA 3'-end.

EMSA gels showing the complex formation of full-length LARP7 (C) with hairpins with various 3'-ends. First panel: HP4 ending by 3 uridines (300-331); second panel: HP4 with a supplementary uridine (300-332); third panel: HP4 with a supplementary adenine. RNA dimers formation is indicated by (*) and complexes of the dimers by (**).

Figure S4: The RNA at a crystal packing interface

(A) The 5-mer RNA (in grey sticks) with the 3'-end uridines bound between LAM (yellow) and RRM1 (orange) and nucleotides U-4 and C-5 in a crystal packing contact with the RRM1 of a neighboring RRM1 (in cyan, residues numbered with *). **(B)** EMSA with RNA M1-HP4 and wild-type (WT) or mutants E130A and F168A versions of the LARP7 La module.

Figure S5: Comparison of LARP7 binding with the 3' hairpin of 7SK and its extended version.

(A) Native gel analysis of complexes (arrows) of HP4 (left) and the 5' extended 262-HP4 RNA (right) with LARP7 full-length (FL, green) or domains: La module (Ndom, orange) or C-terminal domain (Cdom, purple). **(B)** Complex formation as a function of protein concentration (full-length, green; La module, orange; C-terminal domain, purple).

Figure S6. SAXS analysis of the complex of RNA M1-HP4 with the C-terminal domain.

(A) SAXS data analysis. Black line shows the experimental SAXS of a sample of M1-HP4 incubated with the C-terminal domain, and the red line shows the curve obtained from the atomic model of the complex. The logarithm of intensity is displayed as a function of the logarithm of scattering vector q and a residual plot calculated from the relation $R(q) = (I_{exp}(q) - I_{model}(q)) / \sigma_{Iexp(q)}$ is also displayed with the corresponding chi² value. **(B)** The low resolution molecular shape calculated with GASBOR (gray transparent spheres) and the atomic model performed with SASREF showed in two orientations.

Figure S7. Mutational analysis of the C-terminal domain binding to HP4

(A) Alignment of the P65 and P43 sequences with a set of 4 LARP7 sequences in the region of RRM2. Top numbers correspond to the tetrahymena P65 sequence, bottom green numbers to the human LARP7. Specific residues of LARP7 which were mutated are shaded green. **(B)** Our working model in the region of the apical loop of HP4, showing the P65 structure as a purple ribbon. The residues in stick are predicted to be involved in RNA binding, either because they belong to the RNP-2 (purple, Tyr407 in P65, Tyr483 in human LARP7), or correspond to LARP7-specific residues (Tyr513 and Lys517 in P65 align with Lys535 and Asp539 in LARP7, respectively). **(C)** EMSA of the C-terminal domain of LARP7 with M1-HP4, either wild-type (WT) or mutated at the LARP7-specific residues Lys535 and Asp539.

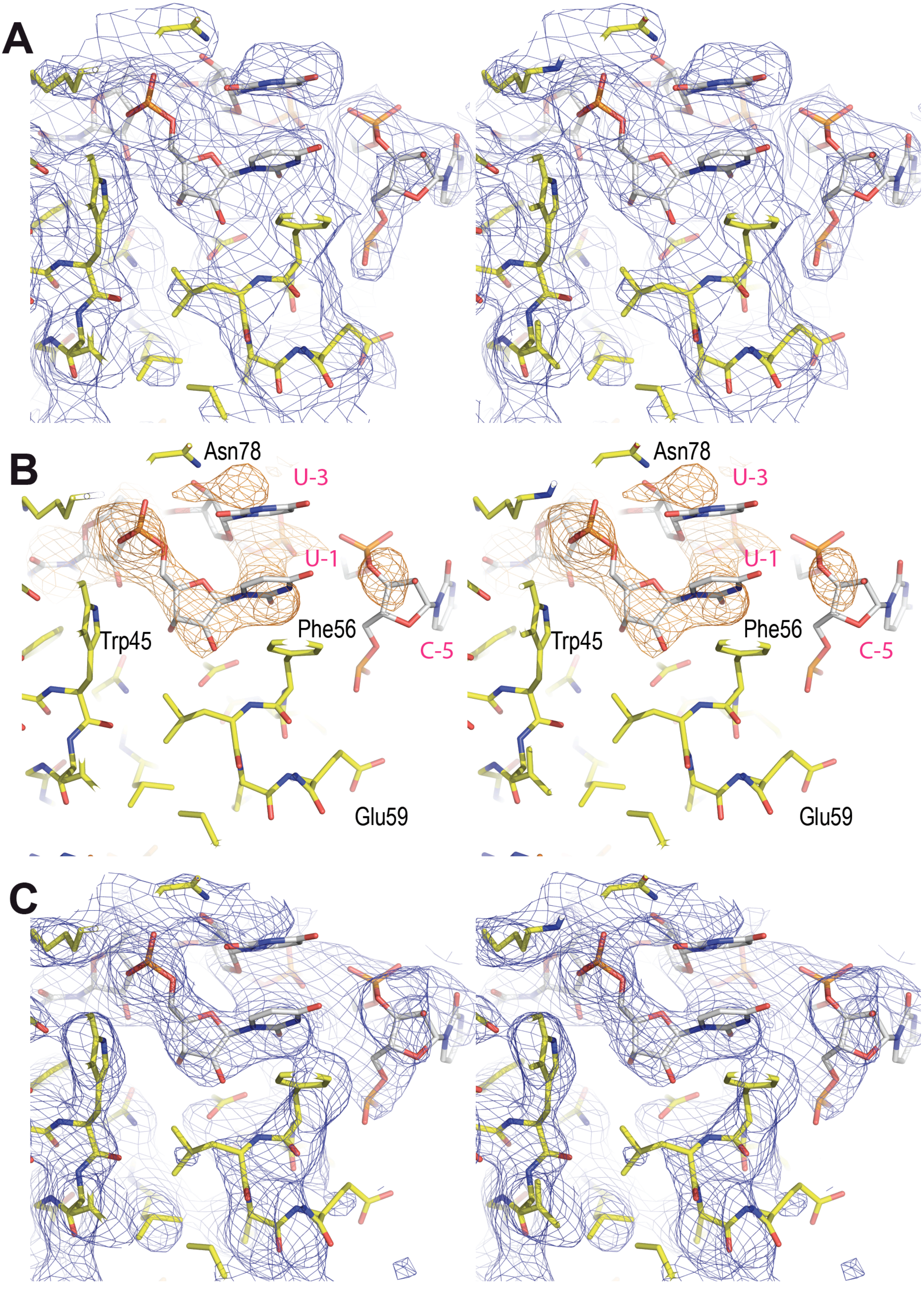

Supplementary Fig. S1

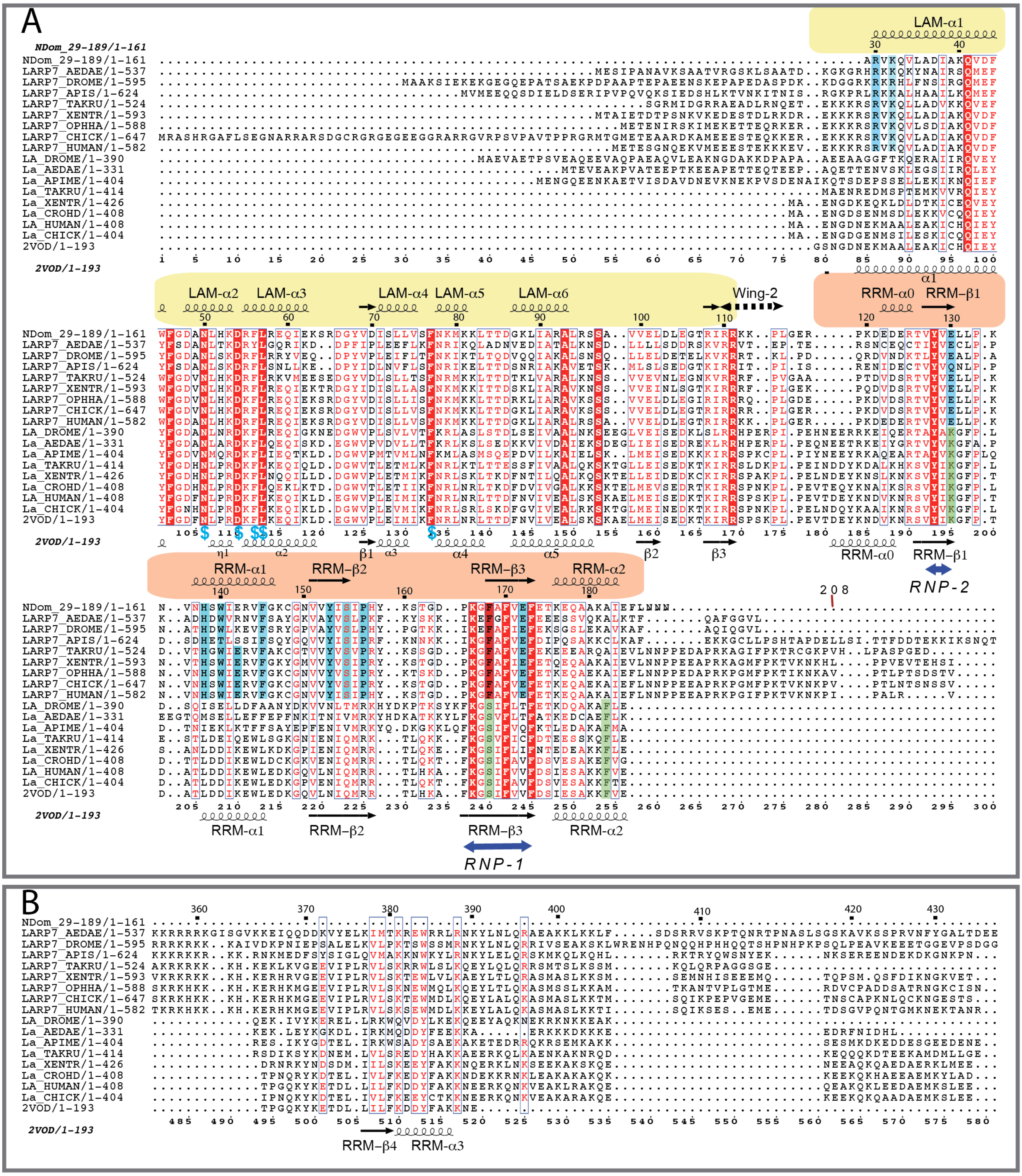

Supplementary Fig. S2

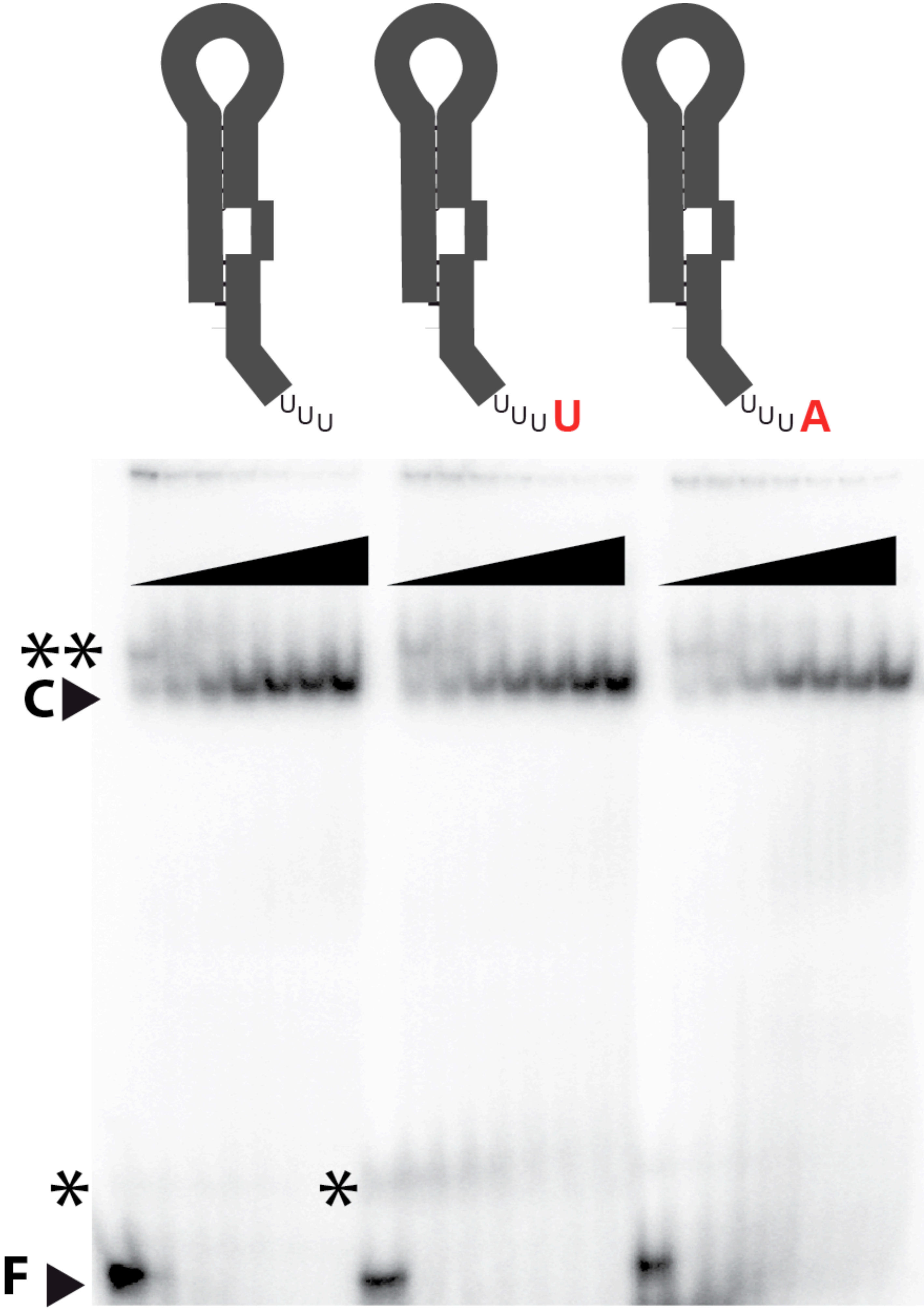

Supplementary Fig. S3

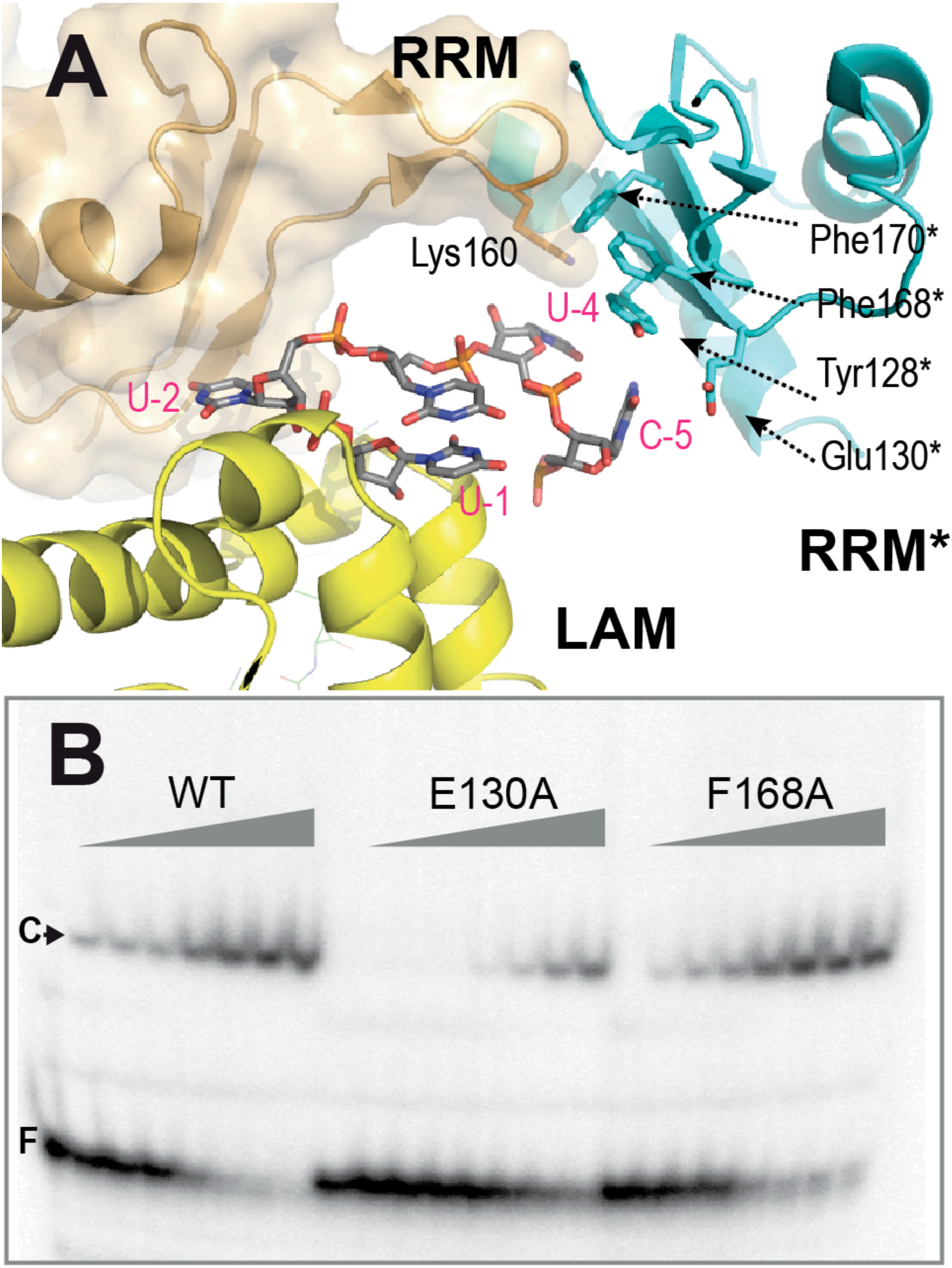

Supplementary Fig. S4

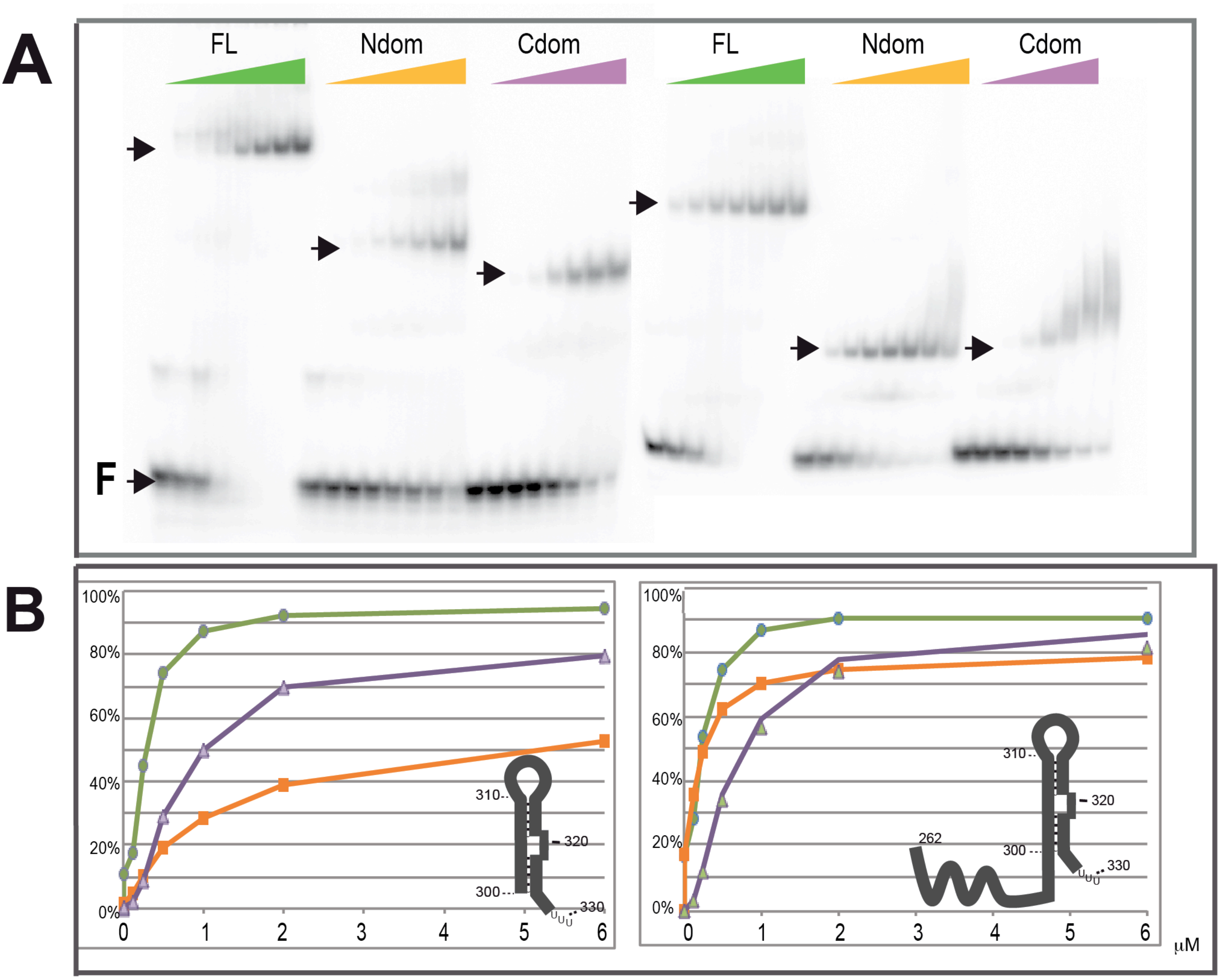

Supplementary Fig. S5

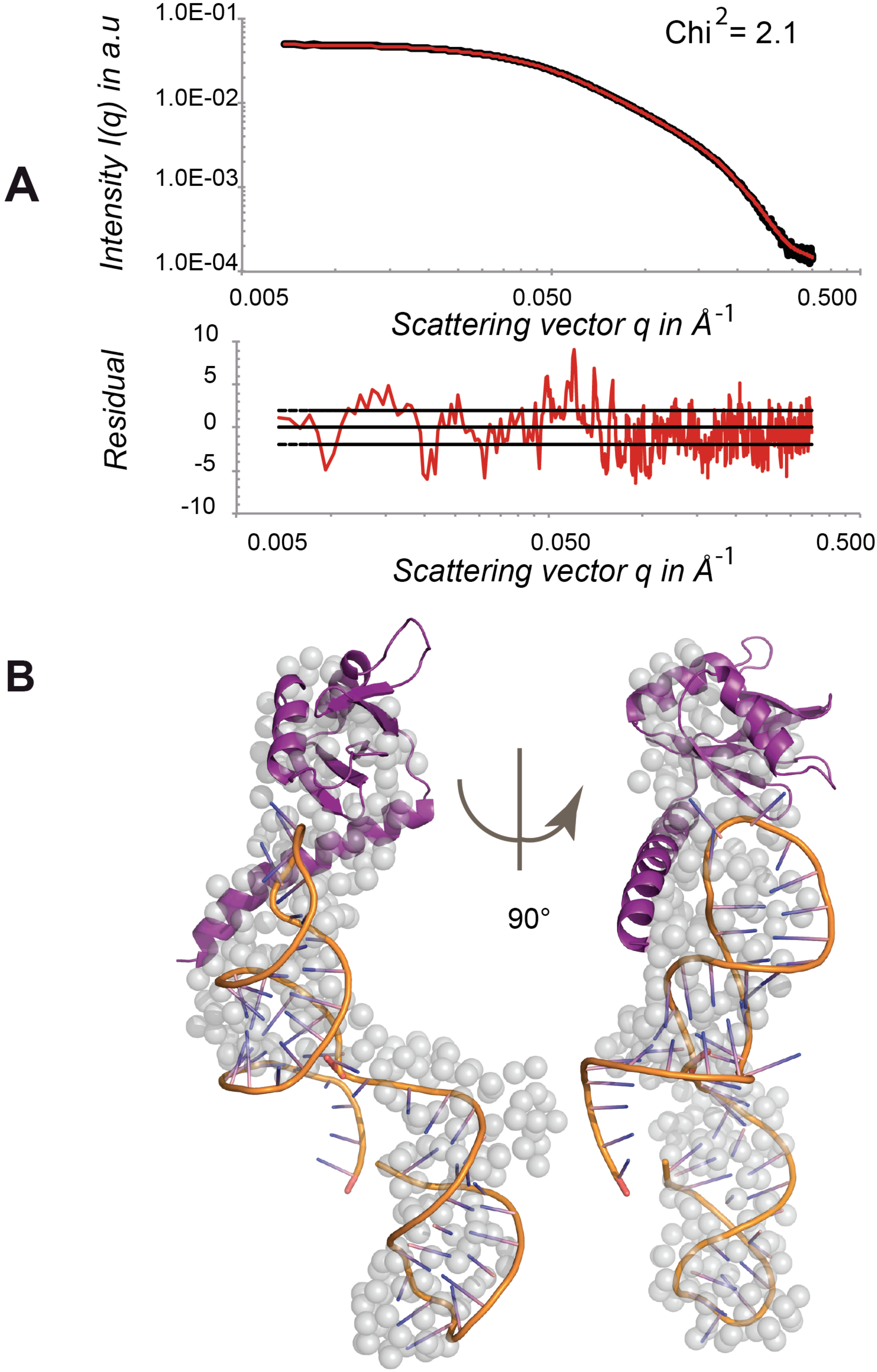

Supplementary Fig. S6

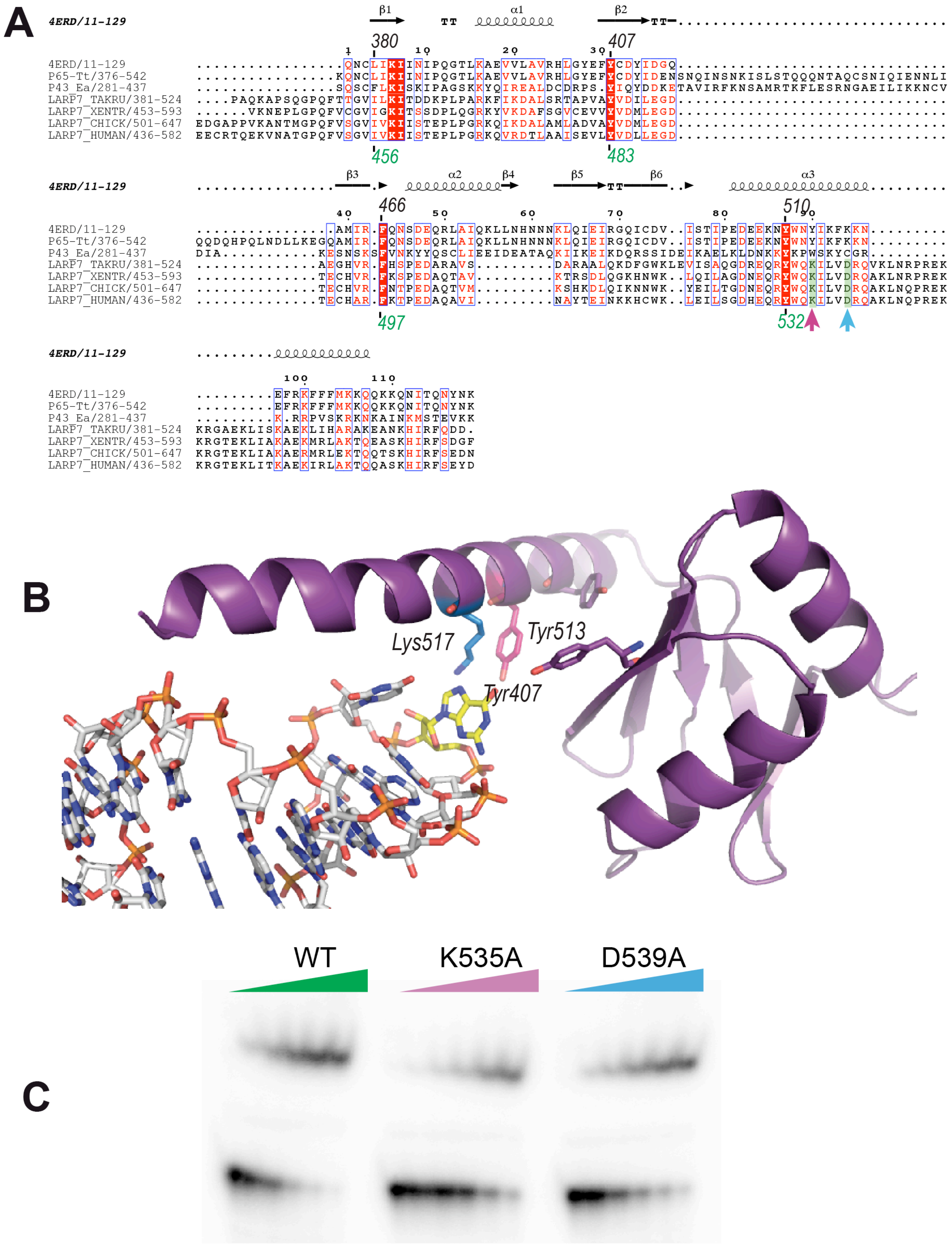

Supplementary Fig. S7
